# Islands within an island: Population genetic structure of the endemic Sardinian newt, *Euproctus platycephalus*

**DOI:** 10.1002/ece3.2665

**Published:** 2017-01-25

**Authors:** Sarah E. Ball, Stefano Bovero, Giuseppe Sotgiu, Giulia Tessa, Claudio Angelini, Jon Bielby, Christopher Durrant, Marco Favelli, Enrico Gazzaniga, Trenton W. J. Garner

**Affiliations:** ^1^ Institute of ZoologyThe Zoological Society of LondonLondonUK; ^2^ Zirichiltaggi S. W. C. Non‐profit Association for Wildlife ConservationSassariItaly; ^3^ Dipartimento di Scienze della Vita e Biologia dei SistemiUniversita degli Studi di TorinoTorinoItaly

**Keywords:** *Euproctus*, genetic structure, Mediterranean island endemic, mitochondria microsatellite discordance

## Abstract

The identification of historic and contemporary barriers to dispersal is central to the conservation of endangered amphibians, but may be hindered by their complex life history and elusive nature. The complementary information generated by mitochondrial (mtDNA) and microsatellite markers generates a valuable tool in elucidating population structure and the impact of habitat fragmentation. We applied this approach to the study of an endangered montane newt, *Euproctus platycephalus*. Endemic to the Mediterranean island of Sardinia, it is threatened by anthropogenic activity, disease, and climate change. We have demonstrated a clear hierarchy of structure across genetically divergent and spatially distinct subpopulations. Divergence between three main mountain regions dominated genetic partitioning with both markers. Mitochondrial phylogeography revealed a deep division dating to ca. 1 million years ago (Mya), isolating the northern region, and further differentiation between the central and southern regions ca. 0.5 Mya, suggesting an association with Pleistocene severe glacial oscillations. Our findings are consistent with a model of southward range expansion during glacial periods, with postglacial range retraction to montane habitat and subsequent genetic isolation. Microsatellite markers revealed further strong population structure, demonstrating significant divergence within the central region, and partial differentiation within the south. The northern population showed reduced genetic diversity. Discordance between mitochondrial and microsatellite markers at this scale indicated a further complexity of population structure, in keeping with male‐biased dispersal and female philopatry. Our study underscores the need to elucidate cryptic population structure in the ecology and conservation strategies for endangered island‐restricted amphibians, especially in the context of disease and climate change.

## 1 Introduction

A global decline in amphibians has been in evidence for more than 25 years (Beebee & Griffiths, 2005). The first Global Amphibian Assessment (GAA) found 32.7% of amphibian species to be categorized as threatened (IUCN 2001), including 427 species listed as being critically endangered, and 122 as possibly extinct (Stuart et al., 2004). Threats such as habitat loss or exploitation are well recognized, and thus potentially reversible, but the declines of nearly half of the species assessed had no clearly identifiable cause. In these cases, epidemic disease on a background of climate change is likely to be contributory (Pounds et al., 2006). However, the study of amphibians is hindered by their complex life history, their typical requirement for both aquatic and terrestrial habitat, and their often elusive nature. Genetic methods provide a powerful tool in the ecology and conservation of such species, for which accurate counts and measurement of dispersal rates are difficult to achieve using classic field ecology approaches (Beebee, 2005; Jehle & Arntzen, 2002). The identification of cryptic diversity is essential to characterize biodiversity and to elucidate underlying evolutionary processes. The assessment and monitoring of genetic variability in threatened populations also provides important information. Small population size and reproductive isolation exacerbate the loss of genetic variability through drift and inbreeding, which may impair the ability of threatened species to respond actively to disease and climate change (Allentoft & O'Brien, 2010; Luquet et al., 2012; Pearman & Garner, 2005).

Historically, mitochondrial DNA (mtDNA) has been the most widely used molecular marker in nonmodel species, largely due to the availability of primers with interspecific cross‐reactivity (Kocher et al., 1989). The study of mtDNA haplotypes has provided important insights into the biogeographical history of amphibians, including the dominance of the Pleistocene glacial cycles in shaping migration and genetic partitioning of temperate amphibian species (reviewed by Hewitt, 2011). The identification of “historical impediments to dispersal” (Avise, 2009) is of relevance to current and future habitat management, especially in the face of climate change. The concept of phylogenetic distinctiveness (Faith, 1992), weighted by conservation urgency, has been proposed for priority‐setting in the conservation of Evolutionarily Distinct and Globally Endangered taxa (Isaac, Turvey, Collen, Waterman, & Baillie, 2007).

Recent advances in genomic sequencing have facilitated the characterization of microsatellite loci in amphibians (Drechsler et al., 2013), previously limited by the complexity of amphibian genomes, and by limited microsatellite cross‐species amplification (Hendrix, Hauswaldt, Veith, & Steinfartz, 2010). In contrast to mtDNA markers, microsatellites provide insight into more recent population genetic processes. Their application thus generates more contemporary information at the level of the individual, allowing the identification of current populations and metapopulations, and the inference of connectivity (Allentoft & O'Brien, 2010) and of barriers to gene flow at a microgeographical scale (Jehle & Arntzen, 2002). The complementary information generated by mtDNA and microsatellites markers thus makes their combined use a valuable tool in population genetics. In addition, the concordance of results from multiple loci reduces the risk of erroneous inferences, as stochastic processes may cause idiosyncratic phylogenetic breaks at a single locus even in the absence of historic barriers to dispersal (Kuo & Avise, 2005; Templeton, 2004).

In this study, we investigate the population structure of the Sardinian brook newt, *Euproctus platycephalus* (Gravenhorst, 1829), a threatened amphibian species (IUCN Red List, 2014) that is difficult to study ecologically, and thus an ideal candidate for this approach (Beebee, 2005). *Euproctus platycephalus* is endemic to Sardinia, a Mediterranean island of area 24,090 km^2^, situated 200 km to the west of the Italian peninsula, from which it is separated by the Tyrrhenian Sea. In marked contrast to the Mediterranean mainland, which has very high amphibian species diversity, Sardinia hosts a limited number of amphibian species (Cox, Chanson, & Stuart, 2006; Cuttelod, Garcia, Malak, Temple, & Katariya, 2009; Grill, Casula, Lecis, & Menken, 2007). Only three genera of Urodela occur on the island, including *E. platycephalus* as the sole salamandrid. *E. platycephalus* is found in the main mountain systems of eastern Sardinia, where it typically breeds in streams at altitudes of 400–1,200 m (Lecis & Norris, 2003; Sindaco, Doria, Mazzetti, & Bernini, 2006; Sotgiu et al., 2010).

The Mediterranean forest landscape of Sardinia (Bacchetta et al., 2009) has been subjected to large‐scale anthropogenic disruption including deforestation, mining, and water abstraction for tourism and agriculture (Corsale, 2011; Rooy & Stumpel, 2003). The island has been the focus of a reforestation initiative (Puddu, Falcucci, & Maiorano, 2012), and much of the surviving habitat is included within protected national and regional parks. However, *Euproctus* habitat continues to be threatened by recurrent severe drought causing summer desiccation of ponds (Pinna, Fonnesu, Sangiorgio, & Basset, 2004). In addition, chytrid infection (*Batrachochytrium dendrobatidis*) has been documented in *E. platycephalus* (Bielby et al., 2013; Bovero et al., 2008; Tessa et al., 2013), an indication of the additional ongoing threat of disease.

The geographical isolation of the genus *Euproctus* is considered to date from the split of the Corsica–Sardinia microplate from the French–Iberian massif, ca. 25 Mya. The subsequent separation of Sardinia from Corsica, ca. 15–9 Mya (Boccaletti et al., 1990), is generally used to infer the timing of the divergence of *E*. *platycephalus* from *Euproctus montanus*, the only other surviving member of the genus, which is endemic to Corsica (Caccone, Milinkovitch, Sbordoni, & Powell, 1997; Carranza & Amat, 2005; Miaud et al., 2009; Steinfartz, Veith, & Tautz, 2000; Zhang, Papenfuss, Wake, Qu, & Wake, 2008). Despite its long isolation, there is no morphological evidence for subspeciation of *E. platycephalus*. It appears remarkably consistent throughout the island, with the exception of a geographical influence on sexual size dimorphism and on timing of life history stages, recently reported by Angelini et al. (2015), which differentiates the southern populations from those of the center and north. Lecis and Norris (2004b), using mitochondrial DNA markers, described intraspecific genetic structure in *E. platycephalus*, broadly partitioning according to the three main mountain regions of Sardinia (Lecis & Norris, 2004b). However, more complex patterns of fragmentation, demographic change, genetic diversity, or gender bias cannot be elucidated by the use of mtDNA as the sole genetic marker (Godinho, Crespo, & Ferrand, 2008).

We have applied the complementary combination of newly characterized microsatellite markers and mtDNA sequencing to investigate the population structure and connectivity of *E. platycephalus*, taking advantage of a comprehensive archive of samples generated during a survey of chytrid infection on the island (Bielby et al., 2013; Bovero et al., 2008). We used coalescence simulation to infer the patterns and timing of divergence and demographic change, based on approximate Bayesian computation (ABC; Beaumont, Zhang, & Balding, 2002). Our results are consistent with both deep genetic divisions and contemporary barriers to gene flow, providing evidence that both historical and more recent factors underpin the current genetic structure. These findings will be of value in the ongoing conservation management of this endangered amphibian, especially in the context of disease and climate change, with the potential of extrapolation to other island endemics.

## 2 Materials and Methods

### 2.1 Samples

*Euproctus platycephalus* samples, comprising skin swabs (Müller, Lenhardt, & Theissinger, 2013), toe clips (Arntzen, Smithson, & Oldham, 1999) or larva tail tips (Polich, Searcy, & Shaffer, 2013), were collected between 2005 and 2012 as part of a survey of *B. dendrobatidis* infection (Bielby et al., 2013; Bovero et al., 2008). DNA was extracted using DNeasy Blood and Tissue kit (Qiagen), with the addition during the tissue lysis step of 0.5‐mm Zirconia/silica beads (BioSpec Products) for swabs and 3‐mm tungsten carbide beads (Qiagen) for toe clips. A total of 227 DNA samples were included in the study, from 13 GPS‐defined sampling sites, spanning the three main mountain ranges of Sardinia to ensure coverage of the geographical range of the species. Results were analyzed for 168 successfully genotyped individuals (detailed in Table 1).

**Table 1:** Demographics of *Euproctus platycephalus* genotyped samples included in the study, showing coordinates and altitude of sampling sites, and year(s) of sampling

Code		Site	Region	Lat N	Long E	Alt m asl	Year	*n*	M	F	nk	L	mtDNA
** 1**	**HP**	**Historical Pool**	North	40.856	9.142	905	2010/12	23	6	5	0	12	10
** 2**	**BIA**	**Fica Bianca**	North	40.842	9.114	983	2007	14	9	2	3	0	10
3	*AFF*	*Affluente Pisciaroni*	North	40.862	9.143	787	2009/11	6	0	0	2	0	4
4	*MO*	*Monte Olia*	North	40.744	9.363	461	2009	1	0	0	1	0	1
** 5**	**BP**	**Bacu su Palu**	Central	40.18	9.559	337	2009	16	1	1	9	5	9
6	*PGC*	*Grotta dei Colombi*	Central	40.172	9.491	597	2010	2	0	0	2	0	0
** 7**	**SES**	**Riu Serra e Scova**	Central	39.974	9.495	854	2010/11	17	8	5	4	0	9
** 8**	**PF**	**Perdas de Fogu**	Central	39.603	9.491	480	2007	15	7	8	0	0	8
** 9**	**MAR**	**Rio Marani**	South	39.348	9.441	162	2007	20	10	8	2	0	10
**10**	**LNR**	**Late Night Return**	South	39.352	9.447	279	2007	14	9	5	0	0	11
**11**	**GUN**	**Rio su Gunventu**	South	39.301	9.406	421	2010/11	15	8	4	1	2	10
12	*MAI*	*Rio Maidopis*	South	39.302	9.404	715	2005	6	4	2	0	0	6
**13**	**CER**	**Rio sa Ceraxa**	South	39.272	9.442	535	2007	19	10	8	1	0	8

The nine main sites used in cluster analysis are shown in bold, and the additional smaller sites in italic. Lat N, latitude north; long E, longitude east; alt m asl, altitude meters above sea level. *n*, number of individuals per site typed with microsatellite markers (total = 168); mtDNA, number of individuals also typed with mitochondrial DNA; M, adult or juvenile male; F, adult or juvenile female; nk, adult or juvenile, gender not known; L, larva; MO, Monte Olia.

### 2.2 Genotyping

#### 2.2.1 Mitochondrial DNA

A D‐loop mtDNA sequence was selected to be comparable with the study of Steinfartz et al. (2000), who estimated a substitution rate of 0.74%–0.86% per My in a phylogeny of D‐loop sequences across *Salamandra*. MtDNA was amplified using primers L‐pro‐ML and E. platy‐H (Steinfartz et al., 2000) in 20 μl volumes with 50–200 ng DNA, 10 μl HotStarTaq Plus mastermix (Qiagen) and 5 μmol/L primers, with initial denaturation 95°C 5 min, 40 cycles of 94°C 30 s, 58°C 90 s, 72°C 90 s, and final extension 72°C 10 min. PCR products were purified by precipitation with 2.5 mol/L NaCl/20% PEG8000 (Teknova Inc). Sanger sequencing in each orientation, using L‐pro‐ML, and an internal primer, SarEu1‐H (Lecis & Norris, 2004b), was undertaken commercially by GATC Biotech (Zurich). Electropherograms were visualized with Sequence Scanner Software 2 (Applied Biosystems).

#### 2.2.2 Microsatellites

Microsatellite markers were developed from *E. platycephalus* sequences by Ecogenics GmbH (Switzerland) using high‐throughput sequencing as described by Abdelkrim, Robertson, Stanton, and Gemmell (2009). In brief, 7 μg of genomic DNA was analyzed on a Roche 454 GS‐FLX platform. Of 48,848 reads with an average length of 294 base pairs (bp), 106 were found to contain a microsatellite insert with a minimum of six repeats for a tri‐ or tetranucleotide unit, or 10 repeats for a dinucleotide unit. Primers were designed by Ecogenics GmbH for 36 microsatellite inserts and tested using 15 individual *E. platycephalus* DNA samples from the Institute of Zoology archive. After further preliminary studies, eight polymorphic loci were selected for genotyping. Loci were amplified in simplex under conditions optimized for each primer pair, as preliminary studies had shown a high incidence of secondary products with multiplex PCR, which impeded allele scoring. PCR was performed in 10 μl volumes with 20–100 ng DNA, 5 μl mastermix (HotStarTaq Plus or Multiplex; Qiagen), 5 μmol/L unlabeled reverse primer and 5 μmol/L fluorophore‐labeled forward primer (Applied Biosystems). Amplification was performed in a G‐Storm GS1 thermal cycler (Gene Technologies), with initial denaturation 95°C 5 min, 45 cycles of 94°C 30 s, 57–59°C 90 s, 72°C 90 s, and final extension 72°C 10 min. Primer sequences and locus‐specific PCR conditions are summarized in Table 2. Amplified products were resolved by capillary electrophoresis on a 3130xl Genetic Analyser (Applied Biosystems) with a LIZ‐500 size standard (Applied Biosystems). Alleles were scored manually, using PeakScanner 1.0 software (Applied Biosystems). Scoring was performed blind to the identification of individual samples to reduce subjective bias. If the allele bands could not be unequivocally differentiated from stutter bands, split bands or nonspecific peaks, the PCR was classed as having failed, and repeated. Genotypes were tested for scoring errors using MICROCHECKER (van Oosterhout, Hutchinson, Wills, & Shipley, 2004).

**Table 2:** Characteristics of the eight microsatellite loci used in the study

Locus	Sequence 5′–3′	PCR	Repeat	*A*	Size bp	*H*_o_	*H*_s_	*H*_t_
EP1	**F:** TGG TGC CTC ATG TGT TCC G	57°	ACAT	18	146–218	0.686	0.665	0.838
	**R:** GTG GCG GAT GGA AGC AAA G							
EP7	**F:** TCC AGG TTG GCT TGG ATG G	57°	ATGT	11	244–285	0.389	0.428	0.705
	**R:** AGA ACA GGC CAC AAC AGA G							
EP8	**F:** TCT TTC TAA GAT TAT GGA ACG CC	57°	TGAA	9	168–204	0.44	0.536	0.692
	R: TTC GCA GTC CCA GAT AGC C							
EP16	F: GCT ATG TAT CTG TGC GTG C	57°	AAAT	10	164–208	0.369	0.405	0.438
	R: GGT TTC ACT GCC CGC TTA C							
EP21	F: GCA CTC CAC TGC CTT TTG G	Multi 58°	ACAT	12	164–215	0.574	0.785	0.87
	R: TAT GAA TTG CCC AGT GCC							
EP29	F: ACG AAT CGG CAG TGT CCA G	59° Mg 3.0	AAT	14	180–231	0.402	0.513	0.758
	R: AGC TTG TGT CAG TCT GGT TTG							
EP31	F: ACT GGA AAA AGC TAA TTG ATA GAT G	57°	AGAT	31	159–299	0.596	0.859	0.924
	R: TTG CTT ATG TAA ATT GGA AAT GTT							
EP33	F: GTG CAC CCA GTC AAT CAC AC	59° Mg 2.5	AATC	12	187–223	0.662	0.726	0.778
	R: ACT GAA TCG CAG ACC CAG							

PCR, primer‐specific PCR conditions including annealing temperature; multi, Qiagen Multiplex mastermix (all others in Qiagen HotStarTaqPlus mastermix); Mg, final concentration of MgCl_2_ (mmol/L) if supplemented. F, forward; R, reverse.*A*, total number of alleles in 168 individuals from 13 sampling locations; size: range of allele sizes; bp, base pairs. Nei's diversity statistics (Nei, 1973) were calculated in FSTAT jackknifed across populations, using nine populations comprising 14–32 individuals, *H*_o,_ observed heterozygosity; *H*_s_, expected heterozygosity under HWE within subpopulations; *H*_t,_ expected heterozygosity in total population.

### 2.3 mtDNA data analysis

Consensus 491‐bp sequences of noncoding D‐loop mtDNA were aligned using ClustalW (Larkin et al., 2007) implemented in BioEdit v7.2.0 (Hall, 1999). Haplotype diversity, nucleotide diversity and genetic differentiation between populations were computed in DNasp v5 (Librado & Rozas, 2009) using 1,000 permutations. Nucleotide substitution models (Nei & Kumar, 2000) were compared in MEGA5 (Tamura et al., 2011). The Tamura three‐parameter model with gamma distribution (T92 + G; Tamura, 1992) and the Hasegawa–Kishino–Yano (HKY) model with gamma distribution and a proportion of invariable sites (Hasegawa, Kishino, & Yano, 1985) gave the lowest values of Akaike information criterion, corrected (AICc) and were used in subsequent analyses as indicated.

Maximum‐likelihood tree topology was inferred in MEGA5, with the inclusion of a homologous D‐loop sequence for *E. montanus* (GenBank EU880316; Zhang et al., 2008). *Triturus cristatus* (GenBank EU880336; Zhang et al., 2008) was used as the outgroup sequence, being within the clade of European newts encompassing *Euproctus* (Weisrock et al., 2006; Zhang et al., 2008), but outside the monophyletic *Euproctus* in‐group (Sanderson & Shaffer, 2002). Topology was tested by bootstrap resampling (1,000 replicates).

Molecular dating was performed in BEAST v2.1.3 (Bouckaert et al., 2014), with mtDNA sequences reduced to haplotypes, and a calibrated Yule tree model. HKY was used as the site substitution model (Hasegawa et al., 1985). A monophyly prior was used to constrain the *E. platycephalus–E. montanus* node to allow for calibration. This had no effect on topology and node support as shown by comparison with a tree without constraints (not shown). To test the assumption of a constant rate of nucleotide substitution, log‐likelihood (lnL) values of trees including with and without molecular clock constraints were compared using MEGA5. The null hypothesis of equal evolutionary rate throughout the tree was not rejected (with clock lnL = −1039.21, without clock lnL = −1023.49, *p* = .1411). This is in keeping with the rate constancy reported in interspecific studies of salamandrids (Caccone et al., 1997; Weisrock et al., 2006). In the absence of independent evidence to inform the calibration of a relaxed clock model (Arbogast, Edwards, Wakeley, Beerli, & Slowinski, 2002), a strict molecular clock was therefore used. For the analysis of population data and in closely related species, as in our study, this can be considered a “good approximate of reality” (dos Reis, Donoghue, & Yang, 2016). An arbitrary prior age of 12 My was specified on the *E. platycephalus–E. montanus* divergence node, selected to reflect the midpoint of the estimated time of separation of Corsica and Sardinia ca. 15–9 Mya (Boccaletti et al., 1990). A normal distribution was used to reflect bidirectional calibration uncertainty (mean 12 My and sigma 1.5, giving a central 97.5% range of 9–15 My). The program was run with 10^8^ Markov chain Monte Carlo cycles, with 20% burn‐in. Chain convergence and measures of effective sample sizes were assessed in Tracer v1.6 (Rambaut, Suchard, Xie, & Drummond, 2014). Files from three independent runs were combined with LogCombiner in BEAST v2.1.3. TreeAnnotator was then used to determine the maximum clade credibility tree. Trees were edited in FigTree v1.4 (Rambaut, 2012).

To further analyze the intraspecific mtDNA genealogy, a haplotype network (Posada & Crandall, 2001) was constructed in NETWORK v4.6.1 (www.fluxus-engineering.com) using the median‐joining method (Bandelt, Forster, & Röhl, 1999). TCS software (Clement, Posada, & Crandall, 2000) was used to implement the parsimony method (Templeton, Crandall, & Sing, 1992), to complement NETWORK results, and to identify the likely ancestral or root haplotype. We estimated evolutionary divergence (*D*_ML_) between the ancestral haplotype and the other haplotype sequences using the Tamura 3‐parameter model in MEGA5 to calculate the number of base substitutions per site between sequences. The rate variation among sites was modeled with a gamma distribution (shape parameter = 0.3). This was used to derive the rate of nucleotide substitution (*r*) and time (*T*) of divergence between haplogroups, according to *T* = *D*_ML_/2*r* (Hughes & Verra, 2001), using values of *T* derived from calibration in BEAST, or a mtDNA D‐loop mean mutation rate (*r*) in salamanders of 0.8%/My, as estimated by Steinfartz et al. (2000).

We investigated potential population size changes in DNAsp v5 (Librado & Rozas, 2009) using mismatch distribution analysis and determining *raggedness r* (Harpending, 1994), D* and F* (Fu & Li, 1993) and *R*_2_ (Ramos‐Onsins & Rozas, 2002) statistics. The observed distribution of pairwise differences was compared with the distribution expected for a model of sudden expansion (Rogers & Harpending, 1992).

### 2.4 Microsatellite data analysis

Microsatellite loci were tested for linkage disequilibrium in FSTAT v 2.9.3.2 (Goudet, 2001). FSTAT was also used to test allele richness, corrected for sample size, and estimators of *F* statistics (Weir & Cockerham, 1984) across loci and populations. Relatedness was estimated as for Queller and Goodnight (1989). The inbreeding coefficient *F* (Frankham, Ballou, & Briscoe, 2010) was estimated using the *inbreeding* function of the adegenet package, version 2.0.0 (Jombart, 2008) implemented in R, version 3.2.2 (R Development Core Team, 2015). A Wilcoxon rank‐sum test, implemented in R, was used to compare the means of estimated *F* values between different regions. Differentiation between populations was tested by pairwise *F*_ST_ in FSTAT, with 1,000 permutations and Bonferroni correction (Rice, 1989). Populations were compared with respect to allele richness and *F* statistics in FSTAT, using 1,000 permutations. An unrooted neighbor‐joining tree (Saitou & Nei, 1987) was generated with the method of Fitch (Fitch & Margoliash, 1967) in PHYLIP v3.695 (Felsenstein, 1993), based on the pairwise microsatellite genetic distance matrix generated in FSTAT.

STRUCTURE v2.3 (Falush, Stephens, & Pritchard, 2003; Pritchard, Stephens, & Donnelly, 2000) was used to infer genetic clustering according to microsatellite allele frequencies. The nine sampling sites containing more than 10 individuals were included, using the admixture model to allow for the possibility of mixed ancestry. Sampling location was included as *locprior* (Hubisz, Falush, Stephens, & Pritchard, 2009). Ten replicate runs were performed for different numbers of clusters (*K*), for *K* = 1–9, with MCMC 1,000,000 after burn‐in 50,000. Mean log‐likelihood values for each value of *K* were computed in Structure Harvester (Earl, 2012), which also derived values of delta*K*, based on the rate of change in log probability for different values of *K*, detecting the uppermost hierarchical level of structure (Evanno, Regnaut, & Goudet, 2005). The highest log‐likelihood posterior probability was seen at *K* = 6 (shown in Section 3). To assign probabilistically the proportion of individual membership to each of the six STRUCTURE‐defined clusters for individuals from the smaller sampling sites not included in the original analysis, STRUCTURE was rerun for *K* = 6 including all samples. Cluster membership coefficients from replicate runs were permuted in CLUMPP (Jakobsson & Rosenberg, 2007).

Hierarchical clustering was further investigated using discriminant analysis of principal components (DAPC; Jombart, Devillard, & Balloux, 2010) in the adegenet package, version 2.0.0 (Jombart, 2008; Jombart & Ahmed, 2011) implemented in R, version 3.2.2 (R Development Core Team, 2015). The *find.clusters* function of adegenet was initially applied to individuals from the nine main sampling sites and then to subgroups of the main sites, grouped according to region. The optimal number of clusters was determined by examining the curves of Bayesian information criterion (BIC) values as a function of the number of clusters (*k*) for each case. The *dapc* function was then applied to the same groupings of sites, using cross‐validation and α‐score functions to determine the optimum number of principal components to retain in each analysis.

### 2.5 Approximate Bayesian computation

In this study, we used ABC implemented in DIYABC version 2.1.0 (Cornuet et al., 2014). The underlying process of DIYABC (Beaumont et al., 2002; Cornuet et al., 2008) is to simulate datasets for scenarios, drawing parameters from prior distributions based on observed genetic structure. The simulated datasets with summary statistics closest to those of the observed dataset are then used to identify the model scenario that best fits the data. While this approach cannot confirm the “right” model and generates broader confidence intervals and less accurate parameter estimates than full‐likelihood methods (Robert, Cornuet, Marin, & Pillai, 2011), it provides a useful basis to assess the likelihood of plausible alternative scenarios. ABC will not distinguish between complicated models if the expected values for the summary statistics do not differ (Wakeley, 2003). We therefore adopted the approach of using competing scenarios to address specific questions and then to generate a final model in which to infer posterior parameters for the model. Replicates of basic scenarios were used to establish the range of core parameters and assess the consistency of results. The timing, duration and magnitude of changes in population size were evaluated in models based on supported scenarios.

The mtDNA dataset was used to investigate the divergence of the northern clade, including screening for bottlenecks, and for the population expansion suggested by mtDNA pairwise nucleotide mismatch analysis, using evolutionary scenarios and priors informed by BEAST and NETWORK results. The microsatellite dataset was used to infer evolutionary processes occurring between the partial central/southern divergence shown on mtDNA haplotype analysis, and the current status based on the hierarchical population structure shown by STRUCTURE and DAPC, and to estimate effective population size and more recent demographic changes. Independent simulations were performed for mtDNA and microsatellite genetic datasets because of observed mtDNA/microsatellite discordance, and results suggesting microsatellite homoplasy (see Section 3).

For mtDNA, the HKY mutation model (Hasegawa et al., 1985) was adopted, with parameters informed by the best fitting model in MEGA5 (proportion of invariant sites 61%, shape of the gamma 0.78). Log prior distributions were used for mean mutation rate and mean coefficient, with default settings other than for the minimum mean mutation rate (set at 1.00E‐8) and for the maximum mean coefficient (set at 50). Individual loci were set to take the same value (=mean). Default settings were used for the microsatellite mutation priors, based on the generalized stepwise model (Di Rienzo et al., 1994; Estoup, Jarne, & Cornuet, 2002). The mutation model default priors are consistent with the microsatellite mutation rate observed in the eastern tiger salamander (Bulut et al., 2009). All loci were treated as a single group. A minimum of 10^5^ simulations was performed per scenario. Scenarios were compared using linear discriminant analysis of summary statistics with logistic regression analysis (Beaumont, 2008; Fagundes et al., 2007) for estimation of posterior probability with 95% confidence intervals (Cornuet et al., 2008). A total of 500 pseudo‐observed datasets (pods) were simulated under each scenario to estimate error and assess confidence in scenario choice (Cornuet, Ravigné, & Estoup, 2010). Parameters were estimated from the posterior parameter distributions of the 1% simulated datasets closest to the observed, using logit transformation. Precision and bias of posterior parameters were assessed using pods with parameter values drawn from the posterior distribution for the relevant scenario. Models were also evaluated for goodness of fit and potential discrepancies (Gelman, Carlin, Stern, & Rubin, 1995), with the inclusion of a range of summary statistics that had not been used for the original simulation process (Cornuet et al., 2010). ABC methods including prior settings and summary statistics used for simulation and for model checking are further detailed in the Supporting information.

## 3 Results

### 3.1 Quality of DNA

Poor amplification, irrespective of the primer set used, was observed in 59 DNA samples (26% of total), all of which had been extracted from swab samples. This failure rate reflects both low yield, and a DNA extraction method which had been optimized for the detection of *B. dendrobatidis* spores. These samples were not included in subsequent analysis.

### 3.2 Mitochondrial DNA

A D‐loop fragment was amplified and sequenced in 96 individuals, including a minimum of eight from each of the nine main sampling sites (Table 1). The final alignment was 491 bp, including 16 variable sites, 13 being parsimony informative. Fifteen different haplotypes were identified, of which six were unique to single individuals.

**Figure 1:**
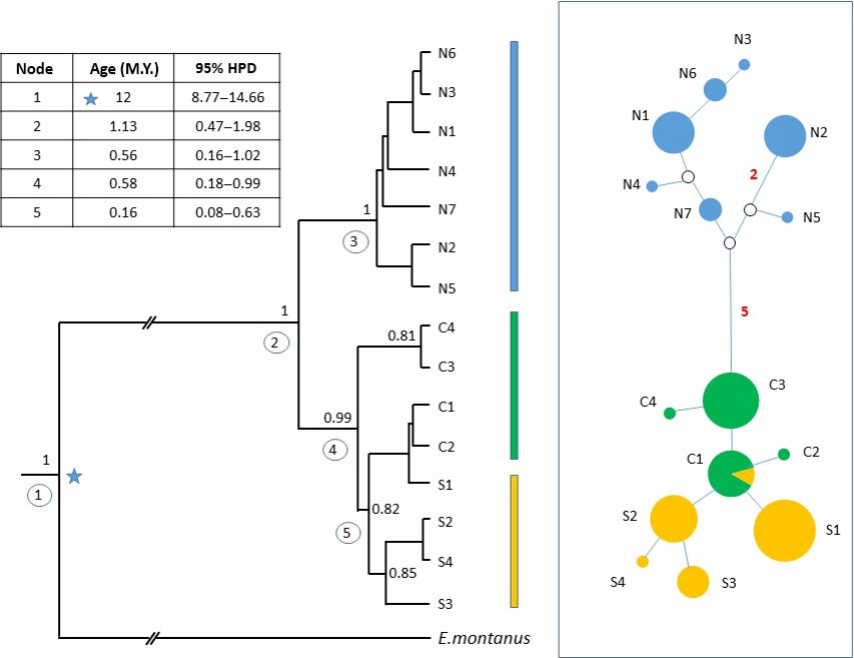
Deep divergence revealed by mtDNA sequencing. Left: Bayesian phylogenetic tree of *Euproctus platycephalus* mtDNA haplotypes implemented in BEAST, using a strict molecular clock model. The tree was rooted with *Triturus cristatus* (not shown on figure for clarity). An arbitrary prior node age of 12 My was specified on the *E. platycephalus–Euproctus montanus* divergence node (starred). Values for posterior probabilities of >0.75 are shown at the relevant nodes. The inset table shows the inferred ages and 95% highest posterior density (HPD) intervals for the numbered nodes. The vertical bars to the right of the tree show clustering of haplotypes into north (blue), central (green), and south (orange) haplogroups. Right: Median‐joining haplotype network, implemented in NETWORK. Circle sizes are proportional to haplotype frequency and colored according to the region of origin of each individual with that haplotype. Numbers represent the number of nucleotide differences between haplotypes, where no number is shown lines between circles represent a single difference. Unrepresented intermediate nodes are shown as an open circle. The *E. montanus* sequence (not shown for clarity) is positioned in the network at 63 mutations from its closest *E. platycephalus* sequence, C2, and thus 64 from C1, the presumed ancestral haplotype

Figure 1 presents the consensus posterior probability phylogenetic tree inferred in BEAST 2 from haplotype sequences, including *E. montanus*, and rooted with *T. cristatus*. Maximum‐likelihood topology and bootstrap support for trees computed with MEGA 5 yielded equivalent results (not shown). All trees showed a very strongly supported node with clear divergence of a clade comprising haplotypes from northern populations from a clade encompassing both south and central regions. Both the northern and the south/central clades showed further divergence with strong nodal support, generating partial separation of central and south haplotypes.

Deep division was also apparent in the median‐joining haplotype network implemented in NETWORK (Figure 1), in which northern haplogroups were separated from central haplogroups by a minimum of five mutations. By contrast, the network showed incomplete resolution between central and southern regions; haplotype C1 was shared between six individuals from central populations, and one from the south. The *E. montanus* sequence was positioned in the network at 63 mutations from the central *E. platycephalus* haplotypes. Equivalent topology was seen in the network yielded using the parsimony method in TCS software (not shown) although the *E. montanus* sequence could not be connected in the same network as *E. platycephalus*.

The table in Figure 1 shows the estimated ages with highest probability density intervals for the well‐supported nodes in the haplotype phylogenetic tree, calibrated according to a 12 Mya split between *E platycephalus* and *E. montanus*. By this approach, the divergence of the northern clade from the central/south was estimated at 1.13 Mya. Further divergence within the north was dated to 0.56 Mya, similar to the onset of divergence within the central and southern haplogroups (0.58 Mya). Based on the maximum‐likelihood genetic distance between *E. montanus* and the C1 haplotype, identified in TCS as the likely ancestral haplotype, the nucleotide substitution rate was calculated to be 0.5% per million years.

**Figure 2:**
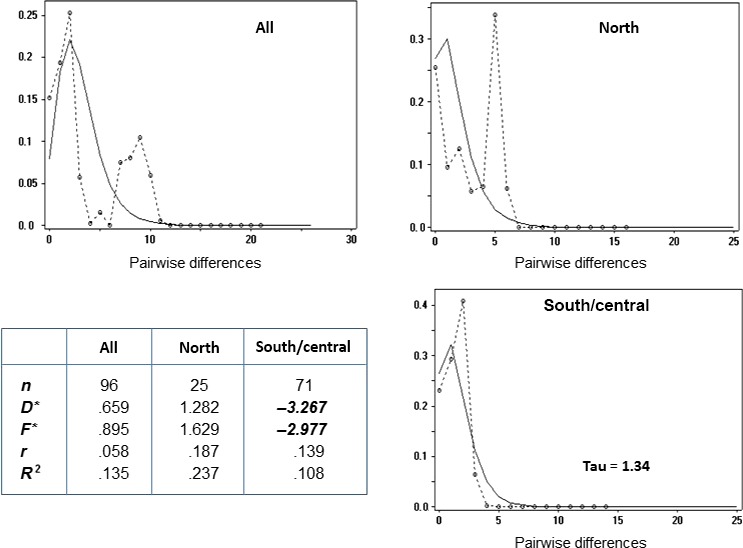
Demographic expansion: pairwise nucleotide mismatch distribution. The graphs show the frequency of observed individual pairwise nucleotide site differences in the mtDNA D‐loop sequence (circles/dotted lines) in comparison with the pattern expected for a sudden expansion model (solid smooth line). The curves for the combined south/central regions show a good visual fit, with a peak at 1–2 nucleotides. The significantly negative values for the neutrality statistics D* and F*, shown in the table, are also consistent with demographic expansion in the south/central region, but not in the northern. all, All individuals; individuals from northern region; south/central, pooled individuals from south and central regions; *n*: number of individuals; D* & F*: Fu's neutrality statistics (Fu & Li, 1993); *R*_2_:*R*_2_ test for neutrality (Ramos‐Onsins & Rozas, 2002); *r*: raggedness index (Harpending, 1994); Statistically significant values are shown in bold italics

Pairwise mismatch distribution analysis of the combined southern and central groups, but not for the northern group, showed a single peak at a mismatch value of 1–2 nucleotides, with a distribution visually similar to that predicted under a sudden expansion model (Figure 2). Values of Fu's D* and F* neutrality statistics (Fu & Li, 1993) for the combined center–south group were significantly negative, in keeping with demographic expansion.

### 3.3 Microsatellites

#### 3.3.1 Diversity

We generated microsatellite genotypes for a total of 168 individuals sampled at 13 GPS‐defined sites. Successful amplification across all eight loci was achieved in 145 individuals (86.4%), with failure to amplify at a single locus in 17 (10.1%), and at 2 loci in 6 (3.5%). There was no association of failure to amplify with individual loci or populations and no significant linkage disequilibrium between any pairs of loci. Polymorphism was apparent at all loci in all populations, with allele numbers ranging from 9 to 31 for individual loci (Table 2). All individual genotypes were different, indicating that no individual had been sampled more than once. No more than two allelic bands were recorded per individual, making it unlikely that skin cells derived more than one individual had been inadvertently sampled, a potential risk associated with the use of external swab samples (Müller et al., 2013). While there was deviation from Hardy–Weinberg equilibrium (HWE; *p* < .001), the results did not suggest the presence of null alleles affecting specific loci or populations (Tables 2 and 3). Simulators of *F* statistics were significantly greater than zero (99% confidence intervals *F*_IT_ .22–.43; *F*_ST_ .095–.316; *F*_IS_ .056–.265), allowing the null hypothesis of panmixia to be rejected. Population structure thus provides the likeliest explanation for the observed deviation from HWE.

**Table 3:** Summary of microsatellite genotyping results in *Euproctus platycephalus*

	Site	Number	*A*_r_	*H*_o_	*H*_s_	*F*_IS_	*F*_ST_
Total	All	168	2.376	0.511	0.613	.166	.185
Regions	North	37	2.693	0.361	0.581	.378	.009
	Central	48	2.764	0.501	0.564	.111	.11
	South	68	3.05	0.597	0.666	.104	.031
Populations							
1	HP	23	2.852	0.385	0.619	.378	.000
2	BIA	14	2.481	0.325	0.52	.376	.001
5	BP	16	2.883	0.515	0.563	.084	.069
7	SES	17	2.798	0.442	0.57	.225	.000
8	PF	15	2.612	0.55	0.56	.019	.000
9	MAR	20	3.068	0.591	0.665	.112	.000
10	LNR	14	2.912	0.597	0.637	.063	.051
11	GUN	15	3.225	0.694	0.683	.000	.002
13	CER	19	3.005	0.526	0.673	.218	.000

Allele richness (*A*_r_), observed (*H*_o_), and expected (*H*_s_) heterozygosity, and fixation statistics (*F*_IS_ and *F*_ST_; Nei & Chesser, 1983) were calculated in FSTAT, with bootstrapping across all eight loci. The top row shows results for the total 168 samples. Results for regions and populations include the nine populations with ≥14 individuals. Negative estimates of *F*_ST_ and FIS are shown as zero.

The two northern populations (HP and BIA) had significantly lower genetic diversity than those from the center or south, as measured by allele richness (*p* < .05), observed heterozygosity (*p* < .01), relatedness (*p* < .05), and *F*_IS_ (*p* < .01). An estimated inbreeding coefficient (*F*) value of >.5, the equivalent of three generations of full‐sib mating (Frankham et al., 2010), was observed in 11 of 37 (29.7%) individuals in the north, six of 48 (12.5%) in the center, and seven of 68 (10.3%) in the south. The mean inbreeding coefficient was also significantly higher for individuals from the northern region than from the south or central regions (mean [*SD*] for north 0.4114 [0.178]; center 0.2882 [0.145]; south 0.2745 [0.143], *p* values for north vs. central and north vs. south <.001). There was no significant difference in genetic diversity between south and central regions. There was no significant difference in relatedness, *F*_IS_ or mean inbreeding coefficient between males (*n* = 41) and females (*n* = 26) pooled from all southern sampling sites (not shown). In the north and central regions, the number of individuals of known gender was insufficient to allow comparison.

#### 3.3.2 Hierarchy of genetic clustering

**Figure 3:**
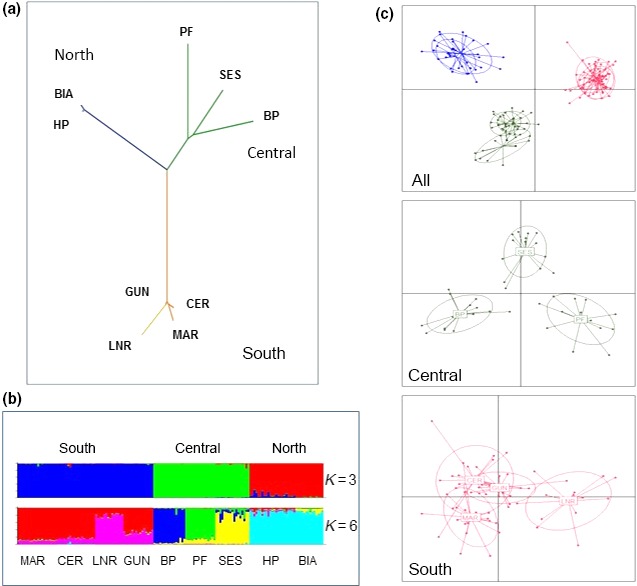
Hierarchical population genetic structure of *Euproctus platycephalus* revealed by microsatellite genotyping. (a) Unrooted neighbor‐joining tree, based on pairwise genetic distance, showing strongly separated regional clades, with intraregional differentiation between the central populations, and partial differentiation between the southern. Branches are colored according to region (blue: north, green: central, orange: south). (b) STRUCTURE results for different values of *K*. The barplot columns represent the Bayesian assignment of individuals to each of *K* genetic clusters, for *K* = 3 and *K* = 6. The probability of membership to each shown as the height of the relevant colored segment. There is clear demarcation between genetic clusters coincident with the spatially defined population groups, consistent with the pairwise *F*_ST_ results. (c) DAPC scatterplots of first two principal components, showing individuals as points, and inertia ellipses for populations defined according to the nine main sampling sites. Points are color‐coded for region of origin (blue: north, green: center, red: south). The top panel shows the results when all nine groups are included, with clear separation according to region. The lower two panels show results from separate analysis of populations from the central and southern sites

To investigate patterns of genetic structuring across the island, the nine sites with the highest number of individuals (range 14–23) were used, comprising two from the north, three central, and four from the south (Table 1). The largest pairwise *F*_ST_ values tended to be between sites from different regions, while the values between the pairs of sites from the same region tended to be lower (Table 4). There was also significant pairwise divergence between the three sites from the central region and in the south between one site (LNR) and the other three sites from the region. A neighbor‐joining tree, based on pairwise differences between the nine populations (Figure 3), illustrates the hierarchy of genetic structure, strongest between regions and between the individual central populations, with a lower level of divergence between the southern populations.

**Table 4:**
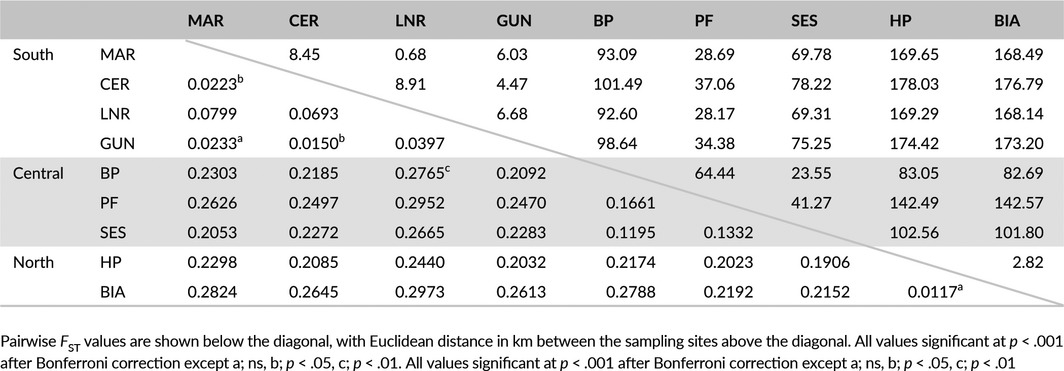
Matrix of pairwise genetic distances between main populations, grouped according to region

**Figure 4:**
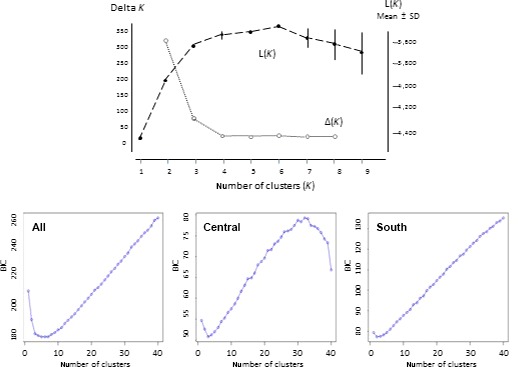
Estimation of number of genetic clusters in microsatellite dataset. Top panel: Variation in posterior probability and delta*K* for different numbers of clusters (*K*) in STRUCTURE. The mean posterior probability *L*(*K*), based on 10 replicate runs, increases sharply up to *K* = 3, reaching a maximum at *K* = 6. At values of *K* above this level, the probability decreases, with higher variance. Delta*K* values (Evanno et al., 2005) most strongly support *K* = 2. Bottom panels: BIC values for different numbers of clusters (*k*) using clustering algorithm in adegenet. The curve for individuals from all nine sampling sites (left) mirrors the *L*(*K*) results. When analyzed separately, the central region (center) shows a clear elbow at *k* = 3, and the southern (right) at *k* = 2

**Figure 5:**
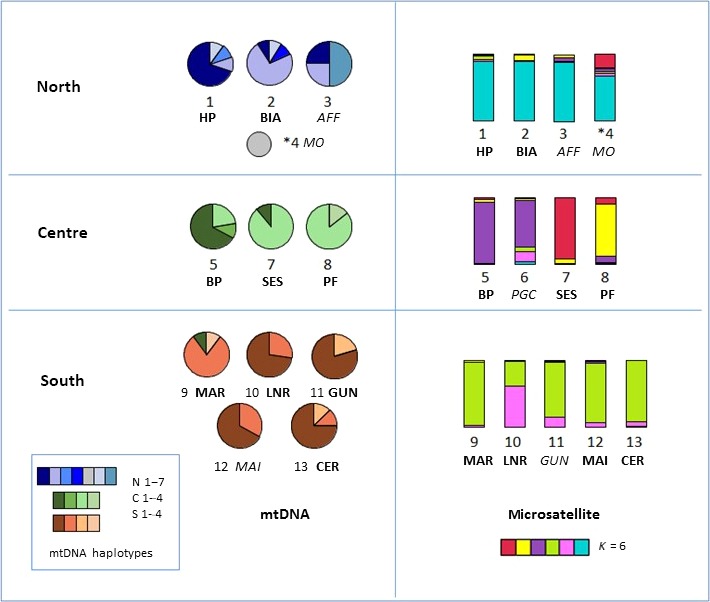
Geographical distribution of mtDNA haplotypes and microsatellite genetic clusters, showing mitochondrial‐microsatellite discordance at intraregional level. Left panel: Pie charts showing patterns of mtDNA haplotype frequency distribution for sampling sites, grouped according to region. The haplotype for the individual from Monte Olia (MO) is shown as a small circle. Right panel: Bar charts showing the proportion of group membership assigned probabilistically to each of *K* = 6 clusters in STRUCTURE for each sampling site. For the single sample from Monte Olia, the individual result is shown (marked *, site 4). The nine major sampling sites are shown in bold and the supplementary sites in italic. See Figure 6 for map of Sardinia showing the locations of the sampling sites

In STRUCTURE, the peak value of deltaK was associated with *K* = 2, but the log‐likelihood posterior probability was highest at *K* = 6 (Figure 4). When the number of clusters was set at *K* = 3, the three main regions were clearly differentiated on barplots (Figure 3). At *K* = 6, the strong regional pattern was still apparent, but there was also clear demarcation between the three central sites, and weaker differentiation between LNR and the other southern locations (Figure 3). Microsatellite STRUCTURE results for the smaller populations at *K* = 6 strongly resembled those of their closest regional neighbors (Figure 5).

An equivalent hierarchical structure was identified using DAPC. When the individuals from the nine main sampling sites were analyzed together, the BIC value declined sharply to a cluster value of 3, then a more gradual decline to *K* = 6 (Figure 4). A DAPC scatterplot of the first two principal components showed clear demarcation between the three regions (Figure 3). When individuals from the three central sites were analyzed separately, the BIC plot showed a sharp elbow at *K* = 3 (Figure 4), and the scatterplot showed clear genetic differentiation between the three sites (Figure 3). Intraregional differentiation was also apparent in the southern samples when analyzed separately, distinguishing individuals from the LNR sampling site from those of the other southern sites. The two northern sampling sites showed no intraregional clustering with DAPC or with STRUCTURE, even when analyzed separately from central and southern sites (not shown).

### 3.4 Discordance between mtDNA and microsatellite markers

Figure 5 presents the frequency distribution of mtDNA haplotypes for the different sampling sites in comparison with the corresponding microsatellite STRUCTURE‐derived bar charts, showing the proportion of membership assigned probabilistically to each of *K* = 6 clusters. Consistent with the strong regional grouping shown in the median‐joining network, mtDNA haplotypes were shared between sites from the same region but not across regions, with the exception of a central haplotype (C1) observed in a single individual from the south. However, striking differences were apparent in the pattern of distribution of mtDNA haplotypes between individual sampling sites from within the same region. This mtDNA/microsatellite discordance is also illustrated by the differences in genetic distance between populations for mtDNA in comparison with microsatellite markers (Table 5).

**Table 5:** Discordance between mtDNA and microsatellites according to region

	mtDNA	Microsatellite
North		
HP:BIA	*	ns
Central		
BP:SES	*	***
BP:PF	*	***
PF:SES	ns	***
South		
MAR:CER	**	*
MAR:GUN	***	ns
MAR:LNR	***	***
CER:GUN	ns	*
CER:LNR	ns	***
GUN:LNR	ns	***

Comparison of levels of significance for pairwise genetic distances between sites with mtDNA and microsatellite markers, showing contrasting patterns of discrepancy between mitochondrial and microsatellite markers according to region. ns; nonsignificant (*p* > .05).

*p < .05; **p < .01; ***p < .001 (after Bonferroni correction).

The most common pattern of discordance was one of divergent mtDNA haplotypes between sites despite almost identical microsatellite patterns, most clearly illustrated by the two main northern sampling sites (HP and BIA; Figure 5). In addition, mtDNA haplotypes for two of the four individuals from the smaller northern site (AFF) were not shared with any of the 20 individuals of the two main sites with the same microsatellite pattern. A unique haplotype was also found in the single individual sampled at the Monte Olia site. A similar pattern of mtDNA/microsatellite discordance was apparent in the southern populations, where the less common S2 haplotype was found to predominate in individuals from MAR, in contrast to the predominant S1 haplotype in the other southern populations despite their having very similar microsatellite patterns.

A different pattern of discordance was apparent in the central region. BP differed from the other two main central sites (SES and PF) with respect to both mtDNA and microsatellite patterns. By contrast, the mtDNA haplotype distribution in SES and PF was very similar, despite strong microsatellite divergence. Unfortunately, there was insufficient DNA to allow sequencing of mtDNA haplotypes in the smaller site (PGC) in the central region.

### 3.5 Approximate Bayesian computation

#### 3.5.1 Simulations using mtDNA datasets

We used ABC of mtDNA datasets to infer the timing and demographics of the divergence of the northern population of *E. platycephalus*. Model scenarios were informed by the mtDNA phylogeny results indicative of an early split of the northern population from an ancestral central (center–south) population, with later onset of divergence of the center and south. The model scenarios used and their posterior probabilities are detailed in Figure S1. As predicted, a scenario of early divergence of the north from an ancestral center–south population, with subsequent divergence of the center and south, was strongly supported over an alternative scenario of simultaneous divergence of all three regions. A third scenario including an unsampled or extinct population was not supported. The timing of the northern divergence, inferred from the posterior distribution of parameters for the supported scenario, was around 3 million generations ago and that of the center–south split around 0.5 million generations ago (Figures S1 and 6).

**Figure 6:**
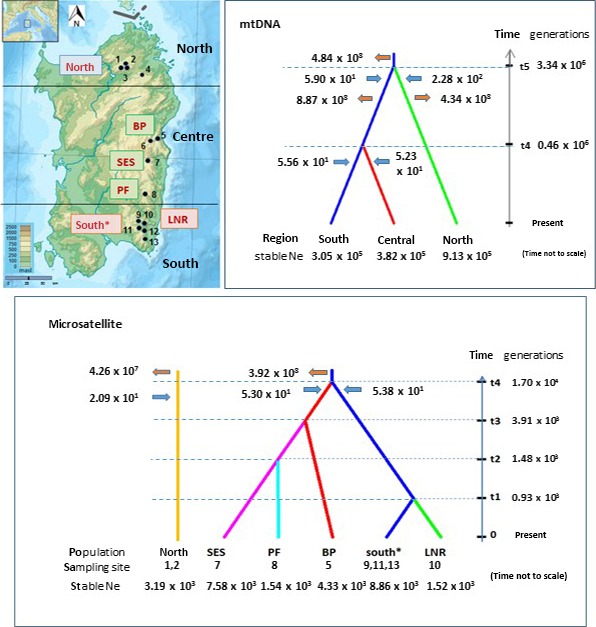
Approximate Bayesian computation scenarios for mtDNA and microsatellite datasets. Supported scenarios for divergence between regions (mtDNA datasets), and between populations (microsatellite datasets). The approximate locations of the sampling sites are shown on the topographic map. Times of divergences (not to scale) are derived from median posterior parameter distributions from replicate simulations (Figures S1D andd S6C). Bottlenecks are indicated by blue arrows and expansions by red arrows (time not to scale). Inferred parameter values for stable *N*_e_ are shown beneath the populations. Inferred values for *N*_e_ during population bottlenecks and expansions are shown next to the appropriate arrow. Note that mtDNA and microsatellite‐derived values are not directly comparable . South*:south populations CER, MAR, GUN

Scenarios incorporating change in effective population size (*N*_e_) were used to screen for bottlenecks at the time of divergence of the northern clade, which might underlie its observed reduction in genetic diversity, as well as for demographic expansion affecting the center–south population, suggested by pairwise mismatch distribution (Figure 2). Methods and results are detailed in the Figs S2 and S3. Results were consistent with a bottleneck affecting not only the northern population, but also the center–south ancestral population around the time of their divergence. There was also support for postbottleneck demographic expansion affecting the diverged northern population as well as the ancestral center–south population, with recovery of both to predivergence levels. ABC results were also in keeping with an equivalent pattern of bottlenecks affecting both center and south populations at their later divergence (Figures 6, S2 and S3).

#### 3.5.2 Simulations using microsatellite datasets

We used microsatellite datasets in DIY ABC to infer events in the center and south, bridging the gap between the partial split between the south and center apparent on mtDNA analysis, and the complete divergence of the central region populations indicated by microsatellite markers. Competing model scenarios and posterior probabilities are shown in Fig. S4.

The supported scenarios are in keeping with early divergence of the south and center and subsequent intraregional divergence. The scenario of a sequential process underlying central intraregional divergence received the highest posterior support (Figures 6 and S4A), consistent with a model of southerly spread and subsequent loss of connectivity. This was endorsed by the comparison of scenarios incorporating admixture, the best‐supported model showing a low level of admixture (inferred proportion 0.189), between the northernmost central population (BP) and the geographically intermediate (SES), and a high level (inferred proportion 0.811) between SES and the southernmost (PF), with minimal support for a scenario of admixture between BP and PF (Fig. S4B). For the origin of LNR, the genetically distinct population in the southern region, the highest posterior support was for a simple split from the rest of the south, with no support for a model of admixture with an unsampled “ghost” population (Fig. S4D). A composite model was generated to incorporate the above supported scenarios (Figure 6), not including admixture for clarity. Parameters derived from posterior distributions of replicate simulations are in keeping with the divergence of the center and south from a common ancestral population 1–2 × 10^4^ generations ago, with differentiation within the central region dating from 3–5 × 10^3^ generations ago.

##### Inclusion of northern region in microsatellite dataset model

The scenario of separation of the north from a center–south common ancestor predictably received the highest posterior support (Fig. S6A), consistent with the mtDNA‐based model. However, the model check showed a less good fit for this scenario (Fig. S6B) than for the composite center–south scenario, without the northern population but otherwise identical (Fig. S6C). In addition, parameters inferred the posterior distributions were inconsistent with the mtDNA‐supported phylogeny in that the divergence of the north from the center–south ancestor appeared to be more recent than that of the center and south. There were also discrepancies apparent in the timing of intraregional divergence of the south and center. (Fig. S6C). These results may be explained by differences in the inferred mtDNA mutation rate in scenarios incorporating the north. While the posterior mutation rate was equivalent for scenarios with only the north microsatellite dataset and for those including the center and south, all scenarios including north with the center and south had a significantly lower inferred mutation rate (Fig. S6D). This is in keeping with the time of coalescence being influenced by the occurrence of homoplasy, in which populations share alleles identical in size but not by descent (Estoup et al., 2002). This is a predictable risk with the microsatellite model of mutation used, especially for ancient events (Cornuet et al., 2010) and distantly related populations (Nauta & Weissing, 1996), and therefore consistent with the historic isolation of the northern population from those in the central and southern regions.

##### Demographic changes inferred from microsatellite datasets

Inferred values for stable *N*_e_ were similar across all six STRUCTURE‐defined populations, in the range 10^3^–10^4^ (Fig. S7B). The supported central/south scenario, not including the northern population, was used as the basic model in which to screen for bottlenecks around the inter‐ and intraregional splits. Posterior support was shown for a bottleneck scenario at the time of the center–south split, affecting both the south and central ancestral populations, but not for bottlenecks around the later intraregional divergence events (Figure S7). To prevent distortion caused by homoplasy, the northern microsatellite dataset was screened separately for demographic change. To ensure comparability with results from the full scenario, this approach was initially applied to the separate south and central mtDNA datasets. Results supported the occurrence of bottlenecks 1–2 × 10^4^ generations ago, affecting both south and central regional populations. This timing is similar to that inferred for the center–south split in the full scenario. A bottleneck affecting the north population was also supported. The inferred timing appeared more recent, around 5 × 10^3^ generations ago, but with confidence intervals overlapping those of the center and south (Fig. S7E).

Individual regional datasets were also used to screen for population expansion. There was good support for demographic expansion events affecting the center and south. Posterior distributions for the expansion models were in keeping with very similar timing of expansion for both center and south regions, predating the divergence of the center and south. For the north dataset, the expansion scenario received lower support, but with a good fit on model checking, including no outliers in distributions of summary statistics. The inferred timing for expansion was equivalent to those for the center and south. Posterior probabilities were close to zero for scenarios with expansion occurring more recently than the center–south split (not shown).

## 4 Discussion

We have demonstrated a clear hierarchy of structure between genetically divergent and spatially distinct subpopulations of the endangered *E. platycephalus* across its range of distribution. With more extensive sampling, we have been able to build on and extend the mtDNA results of Lecis and Norris (2004b). In addition, the combined use of mitochondrial and microsatellite markers has demonstrated further complexity of genetic structure at the microgeographical level and has allowed the application of ABC to address hypotheses relating to the historic and contemporary processes underlying the observed patterns of genetic structure.

### 4.1 mtDNA analysis: deep genetic divisions dating to the Pleistocene

A deep phylogenetic split distancing the northern populations from those from the center and south was the most striking finding in our study with respect to mtDNA haplotypes, in keeping with the results of Lecis and Norris (2004b). This was shown by the strongly supported monophyletic northern clade in the phylogenetic tree and endorsed by the isolation of the northern haplogroups in the mtDNA haplotype networks. Our results indicate that the northern clade of *E. platycephalus* became isolated ca. 1.13 Mya. Divergence between the southern and central populations began later, ca. 0.58 Mya, with very similar timing to the onset of divergence within the northern clade. Bisconti, Canestrelli, Salvi, et al. (2013) found comparable phylogenetic divisions in their mtDNA study of the closely related Corsican endemic *E. montanus*, in which they also used the *E platycephalus–E. montanus* split for node calibration, applying similar caution to avoid overprecision. They estimated that deep intraspecific divergence of *E. montanus* dated to early Pleistocene and terminal haplogroup divergence to late Pleistocene (Bisconti, Canestrelli, Salvi, et al. 2013). Divisions dating to early/mid‐Pleistocene have also been described in the population structure of the Corsican painted frog *Discoglossus montalentii*, another Corsican endemic which occupies a similar montane habitat (Bisconti, Canestrelli & Nascetti, et al., 2013). More distant taxa provide additional evidence of the importance of this period in Tyrrhenian phylogeography. Phylogeny of two Corso‐Sardinian endemic lizards, *Algyroides bedriagae* and *Algyroides fitzingeri*, also shows deep divisions dating to around 1–2 Mya, depending on the substitution rate used for calibration (Salvi, Harris, Bombi, Carretero, & Bologna, 2010; Salvi, Harris, Perera, Bologna, & Carretero, 2011).

### 4.2 Microsatellite analysis: more recent genetic differentiation

There was clear alignment between the historic divisions identified with mtDNA sequencing and the contemporary genetic partitioning between regions shown by microsatellite analysis. The dominance of these deep regional divisions partially obscured structure at a finer geographical scale. However, when the regions were analyzed separately, further differentiation could be discerned. The most striking finding was that of significant divergence within the central region to form three distinct populations, reflecting the higher power of microsatellite datasets to detect more recent patterns of restricted gene flow. The validity of our results is endorsed by their consistency with the different approaches used to detect clustering. The observation of homoplasy does not invalidate these findings, although the pairwise genetic distance between the northern populations and those of the center and south is likely to have been underestimated as a result. This does, however, provide an explanation for why the highest value of deltaK in STRUCTURE was two rather than three, as might have been predicted from regional partitioning.

No significant further differentiation could be discerned within the northern region on microsatellite analysis. This may be explained by the close geographical proximity of the sites in this study, consistent with the high gene flow expected at a microgeographical spatial scale, within the usual dispersal radius of the species (Richardson, Urban, Bolnick, & Skelly, 2014). Our results also suggest extensive gene flow between the sampling sites within the southern region, with the exception of partial but significant divergence between a single site (LNR) and the other southern populations. The ancient divisions between mountain ranges and the clustered sampling of closely neighboring populations across geographically distant sites preclude Mantel testing of isolation by distance (Mantel, 1967; Wright, 1943) across the entire dataset (Meirmans, 2012). Within the common movement range, Euclidean geographical distance is likely to play a less important role than environment in influencing the dispersal of amphibians (Angelone, Kienast, & Holderegger, 2011; Oromi, Amat, Sanuy, & Carranza, 2014; Sexton, Hangartner, & Hoffmann, 2014).

### 4.3 ABC: bridging the gap between mtDNA and microsatellites

Mitochondrial markers provide insight into historic phylogenetic events and microsatellites into contemporary population structure (Wang, 2011). However, there remains in effect a temporal gap between them, illustrated in our study by the lack of information on events occurrence between the partial divergence of the central and south ca. 0.5 Mya, shown on mtDNA analysis, and the striking intraregional differentiation of the central populations, shown on microsatellite analysis. We therefore used ABC to infer intermediary processes, including changes in population size.

Approximate Bayesian computation is approximate by definition, and the accuracy of parameter estimates is lower than for full‐likelihood methods (Robert et al., 2011). Estimates of time parameters are additionally influenced by the mutation model adopted and by estimates of the generation time. In the absence of a unified model of mutation applicable to all microsatellite loci (Balloux & Lugon‐Moulin, 2002; Ellegren, 2004), we adopted the generalized stepwise model (Di Rienzo et al., 1994; Estoup et al., 2002). Estimates of mtDNA and microsatellite mutation rates were broadly compatible with the limited reference ranges available (Bulut et al., 2009; Steinfartz et al., 2000). In this study, our observations of mtDNA–microsatellite discordance and microsatellite homoplasy precluded the incorporation of both mtDNA and microsatellite datasets into the same scenario. However, posterior distributions for mutation model parameters for both mtDNA and microsatellite markers were consistent between supported scenarios of the relevant datasets, with the exception of those affected by homoplasy, allowing the comparison of relative times in generations. Although results for timing and effective population size were not directly comparable across the markers, it was still possible to compare mtDNA and microsatellite scenarios with respect to events around the divergence of the central and southern regions, as mtDNA–microsatellite discordance was only apparent at the finer intraregional scale.

The time to sexual maturity may be used as an approximation of the generation time, defined as the age at which members of given cohort are expected to reproduce (Lande, 1982). The study of Angelini et al. (2015) reported a mean age to sexual maturity of 2–5 years across the island. However, this was influenced by temperature, with a shorter time to sexual maturity in the south than in the north (Angelini et al., 2015). A constant generation time therefore cannot be assumed for our study; during glacial phases, it is likely to have been longer than during interglacial or postglacial phases. This is unlikely to impact on the inferred topology of regional and intraregional divergence, but may further reduce the accuracy of temporal estimates.

The results of screening for changes in population size were less clear‐cut than for the comparison of scenarios of competing topology, but again there was consistency in the timing of bottlenecks and expansion events between full scenarios and separate regional populations, and between mtDNA and microsatellite datasets with respect to changes around the center–south split. This consistency in timing across separate regional populations, with differing patterns of internal population structure, makes it unlikely that the presence of population structure had generated a false signature of population decline (Chikhi, Sousa, Luisi, Goossens, & Beaumont, 2010). A potential disadvantage to the analysis of individual population datasets is that the number of available summary statistics is low, with loss of power of resolution between scenarios, and in model checking. However, the consistency across regions again endorses the plausibility of the results.

#### 4.3.1 Composite biogeographical model

Caveats aside, the ABC approach generated very interesting results, allowing the inference of intermediary events, and providing further insights into historical processes. Analysis of demographic change indicated that the early divergence of the northern clade was likely to have been associated with a sharp population decline affecting both the north and the center–south ancestral populations, rather than the north alone, as might otherwise have been predicted from its lower genetic diversity. Similarly, in contrast to the results of pairwise mismatch analysis, there was support for a postbottleneck expansion affecting the north, as well the center–south ancestral population. The similarity of timing of bottlenecks and expansions relative to the time of divergence is in keeping with a common event impacting on the whole ancestral population, associated with a sharp population decline, and leading to isolation and subsequent expansion of the newly diverged north and center–south populations. This reduces the plausibility of an alternative model of a peripatric origin for the northern population, arising from the migration of founder individuals from the northern margin of the ancestral population.

A common event is also likely to underlie the later divergence of the center and south regions, again indicated by a sharp population decline affecting both diverged populations with equivalent timing relative to the split. Although there was also support for a bottleneck affecting the north, with broadly similar timing, it is not clear whether this reflects a common or an independent process. A predivergence expansion event inferred from microsatellite ABC scenarios for each of the three regions is likely to mirror the rebound expansion inferred from mtDNA scenarios.

According to ABC results, intraregional differentiation occurred independently in the diverged south and central regions. For the central populations, we hypothesize a process of southward expansion, and sequential isolation of the three main central populations, as indicated by the supported topology and admixture scenarios. There was no evidence for rapid expansion following intraregional divergence. It is therefore not clear whether there was gradual full recovery to the prebottleneck population size, followed by a decline to current levels, or a partial recovery followed by population stability, a distinction with clear implications for conservation.

### 4.4 Inference of historical processes

The identification of a strong intraspecific genealogical structure allows inferences to be drawn with respect to the interaction of geography and climate with population structure (Avise, 2009; Avise et al., 1987; Hickerson et al., 2010). Concordance in the temporal pattern of genetic differentiation across diverse geographically codistributed taxa is in keeping with common vicariance events (Avise, 2000; Riddle et al., 2008). The estimated timing of the more terminal haplogroup divergence in the north, and the onset of differentiation between the central and southern regions, fits with that of the severe glacial cycles of early to mid‐Pleistocene (0.9–0.4 Mya; Kahlke et al., 2011; Webb & Bartlein, 1992). *Euproctus platycephalus* is likely to have been relatively protected during glacial maxima, Sardinia being situated south of glaciation permafrost lines (Frenzel, 1992), on a latitude equivalent to that of the Mediterranean peninsula glacial refugia (Hewitt, 2004; Taberlet, Fumagalli, Wust‐Saucy, & Cosson, 1998; reviewed by Schmitt, 2007). As a continental island, Sardinia would have been further protected by marine buffering of climatic oscillations (Cronk, 1997; Médail & Diadema, 2009). There is evidence that it served as a glacial refugium for temperate species (Médail & Diadema, 2009; Vogel, Rumsey, Schneller, Barrett, & Gibby, 1999). However, even within refugia, it is likely that glacial oscillations influenced species distribution and fragmentation by a differential effect on environmental niches and habitats (Cimmaruta, Lucente, & Nascetti, 2015; Gómez & Lunt, 2007; Provan & Bennett, 2008). While glacial maxima tend to drive range retraction for low elevation temperate species, montane species are more likely to expand their range to lowlands during glacial periods (Hewitt, 2011; Stewart, Lister, Barnes, & Dalén, 2010). The effect of glacial periods on the range of *E. platycephalus* may thus have been permissive, promoting connectivity of habitats and facilitating extension of a central population to the southern mountain range of Sardinia, with subsequent postglacial range contraction and restriction to the major mountain systems. Our results relating to the timing of intraregional divergence and the likely southward expansion of the central and southern populations would be consistent with this hypothesis. An equivalent model has been proposed for the dispersal of the sky island salamander *Plethodon ouachitae* across the Ouachita Mountain range during the climatic fluctuations of the Pleistocene (Shepard & Burbrink, 2008). Glacial phase demographic expansion is also considered to underlie the intraspecific genetic structure of the Tyrrhenian tree frog *Hyla sarda*, endemic to Corsica, Sardinia and neighboring islands (Bisconti, Canestrelli, Colangelo, & Nascetti, 2011).

By contrast, the earlier genetic isolation of the northern population of *E. platycephalus* is likely to predate the onset of the severe glacial oscillations. While the Corso‐Sardinian block has generally been considered to have been tectonically stable since around 7 Mya (Mariani, Braitenberg, & Antonioli, 2009), recent data provide evidence for significant reactivation of faults along the East‐Sardinian margin within the time period of the northern isolation (Gaullier et al., 2014; Giresse, Pascucci, Lymer, Gaullier, & Thinon, 2014). Tectonic events thus provide a plausible biogeographical process for the isolation of the northern populations of *E. platycephalus*, whether directly by rifting, or indirectly, for example by marine ingression. Sea is a very effective barrier to the dispersal of freshwater‐dependent salamanders, illustrated by the findings of Hauswaldt et al. (2014) who investigated the divergence of spectacled Salamanders endemic to the Apennine peninsula. The range of their estimate of divergence between *Salamandrina perspicillata* in the north, and the southern *S. terdigitata,* is coincident with that of the recurrent marine submersion of the Volturno–Calore River drainage basin, which lasted into early Pleistocene (Hauswaldt et al., 2014).

Anthropogenic habitat loss and fragmentation over the last century may have influenced the population size and connectivity of *E. platycephalus* across its range. Sardinia has been the site of major hydrological modifications, including the construction of thirty dams from 1920 to 1960, for electricity generation and water supply (Corsale et al., 2011). Neutral genetic divergence may also occur as the indirect result of local adaptation, in which natural selection against immigrants limits gene flow (Wang & Bradburd, 2014).

### 4.5 Mitochondrial‐microsatellite discordance at the intraregional level

In contrast to the observed alignment of genetic divisions between the regions identified by mtDNA and microsatellite markers, at a finer geographical scale, there was mitochondrial‐microsatellite discordance. Within the central region, the occurrence of divergent microsatellite genotypes in the presence of a shared pattern of mtDNA haplotypes can be ascribed to the higher power of microsatellites in detecting more recent obstacles to gene flow. However, within the northern and southern regions, a contrasting pattern of mitochondrial‐microsatellite discordance was apparent. Despite the populations having near‐identical results on microsatellite analysis, and mtDNA haplotypes from the appropriate regional haplogroup, there was significant asymmetry in the frequency distribution of mtDNA haplotypes across sites. Potential explanations for mitochondrial–nuclear discordance in the face of gene flow include mtDNA introgression following hybridization with related taxa, human‐mediated introductions, or selective advantage conferred by specific mtDNA haplotypes (reviewed by Toews & Brelsford, 2012). More than one mechanism may apply; introgression between sister species accounts for mitonuclear discordance in the mole salamander, but with a geographical distribution of mtDNA haplotypes suggestive of a selective advantage in association with levels of precipitation (Denton, Kenyon, Greenwald, & Gibbs, 2014). However, the historic isolation of *E. platycephalus* from closely related species argues against an equivalent mechanism of mitochondrial introgression (Chatfield, Kozak, Fitzpatrick, & Tucker, 2010; Garcia‐Paris, Alcobendas, Buckley, & Wake, 2003). Similarly there here has been no program of human‐mediated release of *E. platycephalus* to support the occurrence of anthropogenic‐mediated hybridization. Environmental differences leading to the selection and fixation of a variant mtDNA at specific sites (Cheviron & Brumfield, 2009; Irwin, 2012) are also unlikely, given the lack of association with a specific mtDNA haplotype and the microgeographical scales involved, especially between the two main northern sites, as well the finding of a similar pattern of discordance in both north and south. This observed pattern of discordance is thus more likely to be related to the matrilineal pattern of inheritance of mtDNA in association with sex‐biased dispersal and demographic asymmetry between sites. The finding of a restricted pattern of mtDNA haplotypes at particular sampling sites could reflect female philopatry to specific pools in a metapopulation pattern of organization that is well recognized in amphibians with a combined aquatic and terrestrial lifestyle (Cushman, 2006; Marsh & Trenham, 2001; Smith & Green, 2005).

Although *E. platycephalus* does not have a physically distinct sex chromosome (Bucci‐Innocenti, Lascialfari, Ragghianti, & Mancino, 1978) to enable direct comparison between patrilineal and matrilineal markers, sex‐biased dispersal may be indirectly inferred from differences in between philopatric and dispersing genders with respect to markers of diversity and relatedness (Cano, Mäkinen, & Merilä, 2008; Goudet, Perrin, & Waser, 2002; Prugnolle & de Meeûs, 2002). We did not find a gender difference in the southern region, but we would predict that, in the presence of high gene flow between the sites, a much higher number of samples would be required to show a difference. There is also a clear need to develop a panel of nuclear markers for *E. platycephalus*, including sex‐linked markers, to further address such questions.

Our study provides an estimate of effective population size, another advantage of the use of microsatellite markers (Luikart, Ryman, Tallmon, Schwartz, & Allendorf, 2010), which provide a more reliable estimate of contemporary population size than mtDNA (Cornuet et al., 2010). These results are remarkably consistent across the different populations, the two smallest relating to the two most recently isolated populations. This is difficult to interpret in the southern population of LNR, which may form part of a metapopulation. The sampling in our study was not restricted to a specific season, and demographic details for the sampling sites are incomplete. It will be interesting to determine whether results vary in relation to the season of sampling, as might occur in the context of a metapopulation structure.

### 4.6 Islands within an island: implications for conservation

A conservation program should ideally retain all ecological processes relevant to the taxon, reflecting site‐specific population structure and terrestrial migration. The combination of a pattern of genetic diversity associated with genetic isolation and adaptive evolution underlies the definition of an evolutionarily significant unit (ESU; Moritz, 2002), proposed by Ryder (1986). While purely genetic methods to define units of conservation below the level of the species are limited in scope (May, Medley, Johnson, & Hoffman, 2011), in the context of cryptic diversity it is otherwise difficult to achieve the aim of ensuring preservation of both adaptive and evolutionary processes across the whole range of the species.

In this study of *E. platycephalus*, the northern region best fulfills the criteria for an ESU, being geographically discrete, and showing reciprocal monophyly for mtDNA (Moritz, 1994), as first indicated by the study of Lecis and Norris (2004b). We have now demonstrated concordant divergence with respect to both nuclear and mitochondrial markers, a further criterion for the stringent classification of an ESU (Moritz, 1994). The importance of the northern region in conservation efforts is additionally highlighted by our finding of a significant reduction in microsatellite genetic diversity and a high level of estimated inbreeding in the northern populations in comparison with the other groups. This loss of genetic variability is likely to be genomewide; there is generally correlated loss between microsatellites, representing neutral genomic markers, and MHC variation (Sutton, Nakagawa, Robertson, & Jamieson, 2011). This reduced diversity may result from the historic isolation and recurrent bottlenecks affecting the northern region, exacerbated by low dispersal rates and site philopatry. Population declines caused by habitat loss and other threats may result in continuing genetic erosion, with potential ecological implications (Hughes, Inouye, Johnson, Underwood, & Vellend, 2008). This is of particular relevance to the higher risk in the north of infection with the chytrid *B. dendrobatidis* (Bielby et al., 2013; Longcore, Pessier, & Nichols, 1999; Tessa et al., 2013). A screening program across Sardinia from 2006 to 2010 showed the prevalence of chytrid infection to be concentrated in populations from the Limbara mountain range in the north of the island (Bielby et al., 2013). This could be an indirect effect of the environment, explained by the climatic requirements of the causative fungal agent (Piotrowski, Annis, & Longcore, 2004; Ron, 2005). However, low genetic diversity may in itself render the northern populations less resilient to infection (Allentoft & O'Brien, 2010; Luquet et al., 2012; Pearman & Garner, 2005). A positive genetic‐fitness correlation has been reported in the majority of studies of amphibians, although there may be a reporting bias (reviewed by Allentoft & O'Brien, 2010). Genomic diversity is likely to be especially important in population recovery. In a manipulative field experiment in which sea grass was exposed to near lethal water warming, microsatellite diversity was associated with enhanced recovery by a process of genotypic complementarity, rather than by the selection of robust genotypes (Reusch, Ehlers, Hämmerli, & Worm, 2005).

The concept of ecological and genetic exchangeability within units of conservation (Crandall, Bininda‐Emonds, Mace, & Wayne, 2000) is especially applicable to the management of isolated declining populations, for which translocation or genetic rescue may be considered. In the absence of gene flow, new variation can only occur in isolated populations as a result of mutation, providing the rationale for genetic rescue in conservation. The risk of outbreeding depression is highest when immigrants are “geographically distinct and genetically divergent” relative to the inbred population (Whiteley, Fitzpatrick, Funk, & Tallmon, 2015). Frankham et al. (2011) found that risk factors in outbreeding depression included a lack of gene exchange in the past 500 years between collection and restoration sites. Screening for such risk factors forms an important part of the management of genetic rescue attempts (Frankham, 2015). The need for caution extends to ex situ conservation activity; careful documentation of the geographical origin of individuals used to establish breeding colonies is essential. While the central and southern regions do not meet the stringent criteria to be classified as independent ESUs, the deep genetic divisions we have identified suggest that they too should not be considered genetically interchangeable. The likely metapopulation organization of the southern populations suggests that they could be treated as a single management unit, despite the apparent divergence of the LNR population. However, the issue of niche exchangeability is more difficult with respect to the three central populations, endorsing the need to define conservation units using both genetics and ecology (May et al., 2011).

### 4.7 Current and future status

Although *E. platycephalus* is protected under Italian Regional Law and the EU Natural Habitats Directive (92/43/EEC), and much of its habitat is now included in Sardinian National and Regional Parks, the consequences of historical fragmentation of habitat and loss of connectivity between component parts of metapopulations may already be critical (Cushman, 2006; Ficetola & De Bernardi, 2004; Marsh & Trenham, 2001). An effect of the warmer and semiarid habitat of the southern populations is already apparent in their smaller size and earlier sexual maturity (Angelini et al., 2015). For the south in particular, continuing loss of ponds through water abstraction and drought remains a threat which is likely to be exacerbated by climate change. It will be necessary to continue to monitor the distribution of *E. platycephalus* in association with measurements of habitat quality, including ambient and water temperature (Lecis & Norris, 2003, 2004a). Genetic monitoring and further elucidation of the population structure and the impact of anthropogenic and geographical barriers to dispersal will play an important part in conservation of this endangered species (Manel, Schwartz, Luikart, & Taberlet, 2003; Storfer, Murphy, Spear, Holderegger, & Waits, 2010).

Our study has demonstrated a complex hierarchical genetic structure in *E. platycephalus*, with a degree of microendemicity that might not otherwise have been anticipated. There remains a still unexplored complexity of metapopulation structure, including sex‐biased philopatry and migration, indicated by our combined use of microsatellite and mtDNA markers. The statement that “any model that endeavours to explain island‐wide patterns must necessarily be complex,” made in reference to the distribution of herpetofauna in Madagascar (Brown, Cameron, Yoder, & Vences, 2014), is equally applicable to Sardinia, a continental island both ten times younger and twenty times smaller.

## Conflict of Interest

None declared.

## Data Accessibility

Microsatellite genotype dataset: Data available from the Dryad Digital Repository: http://dx.doi.org/10.5061/dryad.b60c1Sequence data: GenBank accession numbers KY172000–KY172014.
