# Supplementary material for: Islands within an island: Population genetic structure of the endemic Sardinian newt, Euproctus platycephalus
Source: Ecol Evol. 2017 Jan 25;7(4):1190–211. doi: 10.1002/ece3.2665 (PMC5306002; doi:10.1002/ece3.2665)
Supplement: Supplementary file 1 [file ECE3-7-1190-s001.pdf]

Figure S1

A

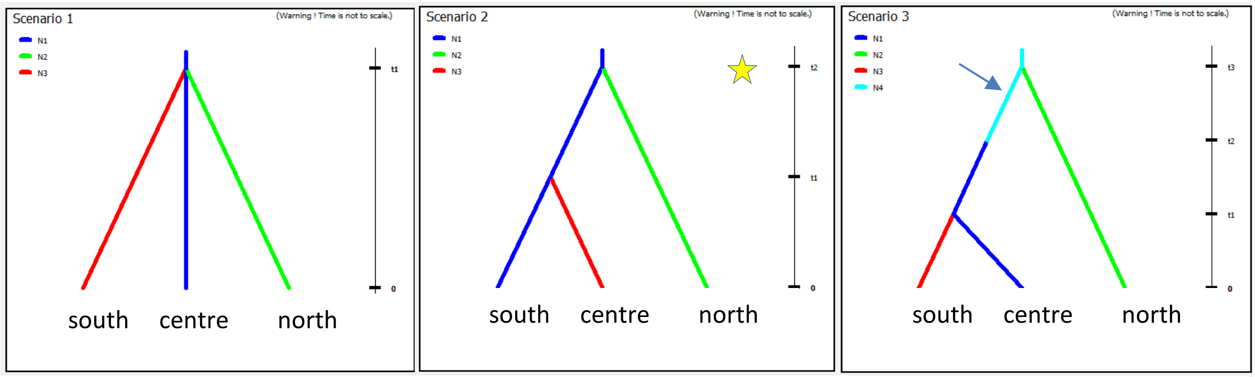

B

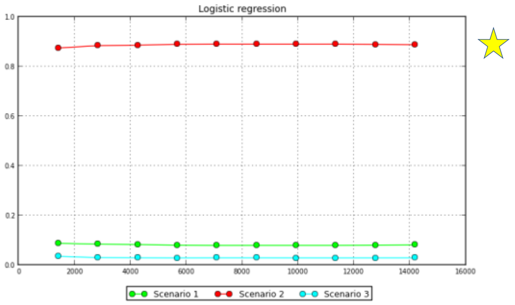

C

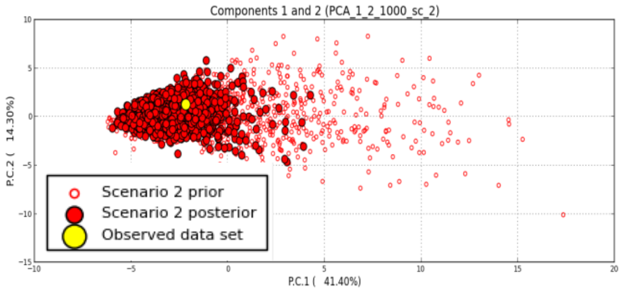

confidence in scenario choice

error	type I	0.093
	type II	0.043

Outliers	total summary stats = 39				
< 0.05	*	0	> 0.95	*	1
	**	0		**	0
	***	0		***	0

D

	event	mean (95% CI) (x10^6 generations)	n
t2	divergence of north	3.34 (2.88 - 3.80)	8
t1	divergence between south and central	0.46 (0.29 - 0.64)	8

E

**Parameter**	**Prior**	**Posterior**				**Measure of performance**					
		**median**	**mode**	**q050**	**q950**	**true value**	**median**	**mode**	**RRMISE**	**RMedAd**	**Factor 2**
N1 (centre)	U [10^2,10^6]	4.61 e+5	3.82 e+5	1.15 e+5	9.12 e+5	4.802 e+5	4.36 e+5	3.853 e+5	1.754	0.52	0.77
N2 (north)	U [10^2,10^6]	7.54 e+5	9.13 e+5	3.76 e+5	9.76 e+5	7.344 e+5	5.971 e+5	6.350 e+5	0.507	0.309	0.876
N3 (south)	U [10^2,10^6]	3.95 e+5	3.05 e+5	8.9 e+4	8.69 e+5	4.355 e+5	4.295 e+5	3.800 e+5	1.636	0.543	0.758
t1	U [10^2,10^6]	2.99 e+5	9.07 e+4	4.99 e+4	8.63 e+5	3.406 e+5	3.543 e+5	2.651 e+5	6.477	0.626	0.69
t2	U [10^2,10^7]	3.20 e+6	2.23 e+6	1.20 e+6	7.16 e+6	3.44 e+6	3.672 e+6	3.182 e+6	1.053	0.44	0.854
μ-seq	standard	1.74 e-8	1.38 e-8	1.10 e-8	3.54 e-8	1.989 e-8	1.924 e-8	1.572 e-8	0.663	0.287	0.946
K-seq	standard	3.62 e+0	6.50 e-2	8.96 e-2	4.16 e+1	1.115 e+1	2.181 e+0	5.551 e-2	57.29	0.944	0.196
