# Supplementary material for: Islands within an island: Population genetic structure of the endemic Sardinian newt, Euproctus platycephalus
Source: Ecol Evol. 2017 Jan 25;7(4):1190–211. doi: 10.1002/ece3.2665 (PMC5306002; doi:10.1002/ece3.2665)
Supplement: Supplementary file 2 [file ECE3-7-1190-s002.pdf]

Figure S2

A

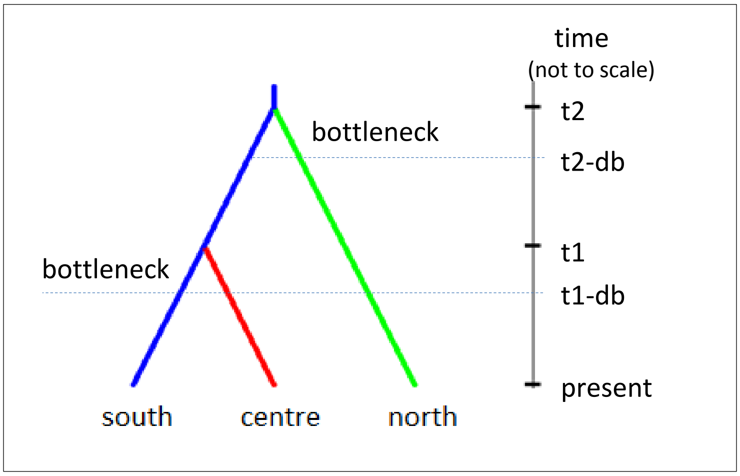

B

time	event	population affected	outliers (total stats=39)					
			< 0.05			> 0.95		
			*	**	***	*	**	***
**t2**	divergence of north from centre-south	north	0	0	0	2	0	0
		centre-south	0	0	0	1	0	0
		north and centre-south	0	0	0	1	0	0
**t1**	divergence of south and central	centre	0	0	0	1	0	0
		south	0	0	0	1	0	0
		centre and south	0	0	0	2	0	0

C

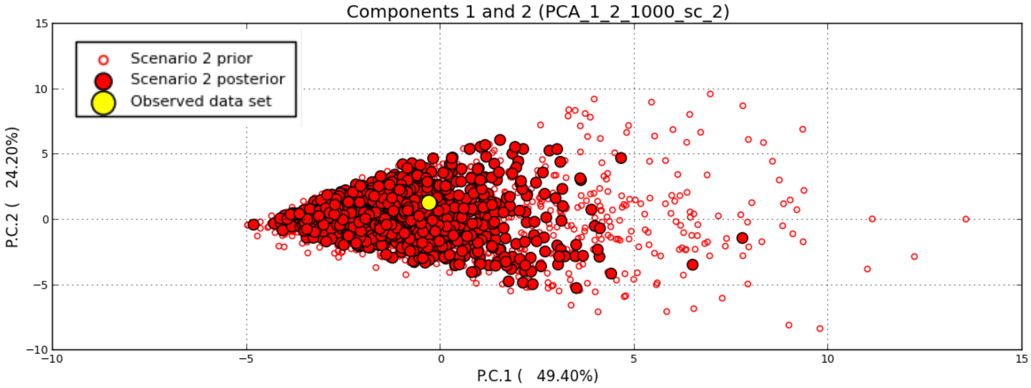

D

population	time (x10^6)		n
	mean	se	
north	6.16	0.17	4
south-central	5.75	0.481	4

prior
U[10^3,10^7]

Bottleneck
Wilcoxon test
W=11
P value=0.4857

E

population	time (x10^6)		n
	mean	se	
north	2.10	0.12	3
south-central	2.21	0.09	3

prior
U[10^3,10^7]

Expansion
Wilcoxon test
W=3
P value=0. 7
