# Supplementary material for: Islands within an island: Population genetic structure of the endemic Sardinian newt, Euproctus platycephalus
Source: Ecol Evol. 2017 Jan 25;7(4):1190–211. doi: 10.1002/ece3.2665 (PMC5306002; doi:10.1002/ece3.2665)
Supplement: Supplementary file 3 [file ECE3-7-1190-s003.pdf]

Figure S3

A

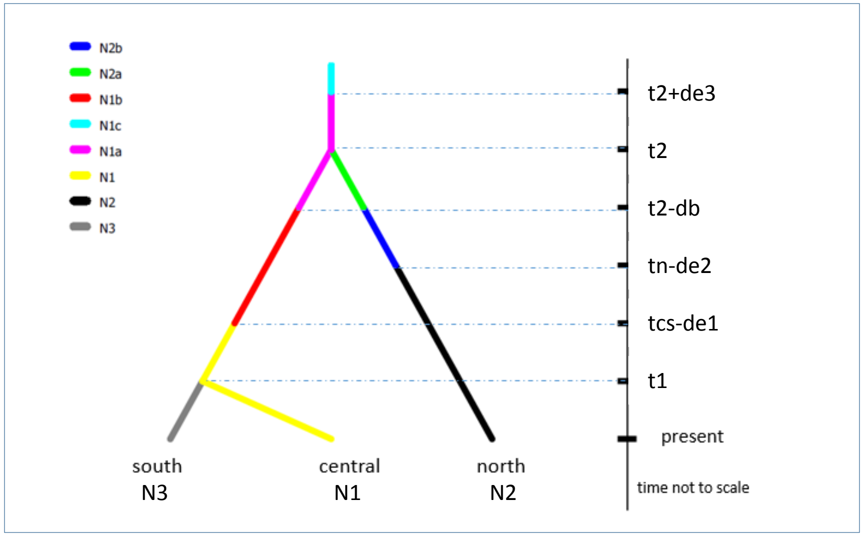

B

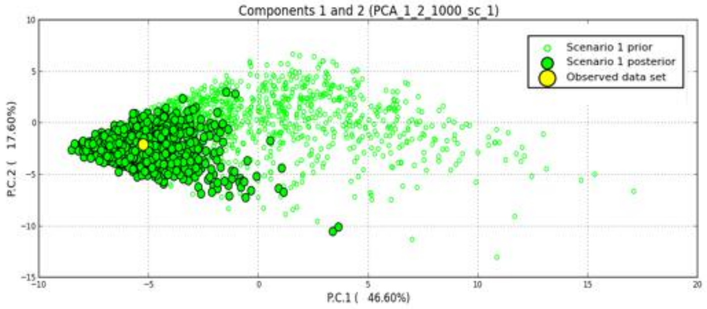

**Outliers**			total summary statistics = 39		
< 0.05	*	0	> 0.95	*	0
	**	0		**	0
	***	0		***	0

C

	parameter	type	prior	posterior				measure of performance					
				median	mode	q050	q950	true value	median	mode	RRMISE	RMedAd	Factor 2
**Ne centre**	N1	N	U $[10^3,10^6]$	5.57 e+5	4.54 e+5	1.70 e+5	9.42 e+5	5.362 e+5	4.507 e+5	4.015 e+5	3.063	0.457	0.822
**Ne north**	N2	N	U $[10^3,10^7]$	1.08 e+6	8.47 e+5	4.29 e+5	2.93 e+6	1.371 e+6	1.384 e+6	1.084 e+6	1.126	0.482	0.784
**Ne south**	N3	N	U $[10^3,10^6]$	5.27 e+5	4.75 e+5	1.58 e+5	9.46 e+5	5.515 e+5	4.871 e+5	4.631 e+5	1.06	0.456	0.862
**split centre/south**	t1	T	U $[10^3,6x10^5]$	2.70 e+5	1.63 e+05	6.10 e+4	5.56 e+5	2.835 e+5	2.745 e+5	2.472 e+5	2.32	0.528	0.806
**split north/ cs**	t2	T	4 $x10^6$ fixed										
**expansion cs**	tcs	T	U $[6.2x10^5,3.8x10^6]$	2.27 e+6	2.32 e+6	4.81 e+5	3.62 e+6	2.231 e+6	2.207 e+6	2.316 e+6	0.813	0.377	0.9
	de1	T	U $[10 ,10^3]$	4.53 e+2	4.89 e+1	4.89 e+1	9.48 e+2	4.783 e+2	5.035 e+2	4.789 e+2	5.027	0.542	0.706
	N1b	N	U $[10^6,10^9]$	4.87 e+8	1.56 e+8	4.81 e+7	9.60 e+8	5.011 e+8	4.960 e+8	4.84 e+8	7.525	0.519	0.738
**expansion north**	tn	T	U $[6.2x10^5,3.8x10^6]$	2.17 e+6	1.13 e+6	8.12 e+05	3.60 e+6	2.253 e+6	2.251 e+6	2.404 e+6	0.816	0.371	0.884
	de2	T	U $[10,10^4]$	4.81 e+3	8.08 e+2	4.70 e+2	9.58 e+3	5.075 e+3	4.980 e+3	4.789 e+3	7.047	0.533	0.7
	N2b	N	U $[10^6,10^9]$	4.34 e+8	2.14 e+7	4.11 e+7	9.49 e+8	4.621 e+8	4.954 e+8	4.516 e+8	18.251	0.594	0.644
**early bottleneck**	db	T	U $[10,10^3]$	4.81 e+2	3.84 e+2	5.40 e+1	9.47 e+2	4.778 e+2	5.061 e+2	5.088 e+2	6.904	0.556	0.692
	N1a	N	U $[10,10^2]$	5.90 e+1	9.24 e+1	1.56 e+1	9.63 e+1	5.839 e+1	5.400 e+1	5.010 e+1	1.244	0.447	0.826
	N2a	N	U $[10, 5x10^2]$	2.28 e+2	8.03 e+1	2.90 e+1	4.69 e+2	2.305 e+2	2.590 e+2	2.739 e+2	3.853	0.573	0.698
**pre-split north/cs**	de3	T	U $[10,10^3]$	5.16 e+2	6.83 e+1	6.89 e+1	9.54 e+2	5.328 e+2	5.132 e+2	5.542 e+2	3.305	0.494	0.71
	N1c	N	U $[10^6,10^9]$	4.84 e+8	2.12 e+8	4.46 e+7	9.49 e+8	4.481 e+8	4.990 e+8	4.869 e+8	16.108	0.584	0.654
**mutation model**	µseq	M	standard	1.43 e-8	1.22 e-8	1.06 e-8	2.38 e-8	1.523 e-8	1.751 e-8	1.535 e-8	0.576	0.228	0.968
	K1seq	M	standard	2.75 e+0	5.49 e-2	7.98 e-2	3.92 e+1	1.058 e+1	2.905 e+0	2.602 e-1	67.493	0.947	0.22
