# Supplementary material for: Islands within an island: Population genetic structure of the endemic Sardinian newt, Euproctus platycephalus
Source: Ecol Evol. 2017 Jan 25;7(4):1190–211. doi: 10.1002/ece3.2665 (PMC5306002; doi:10.1002/ece3.2665)
Supplement: Supplementary file 4 [file ECE3-7-1190-s004.pdf]

Figure S4

A

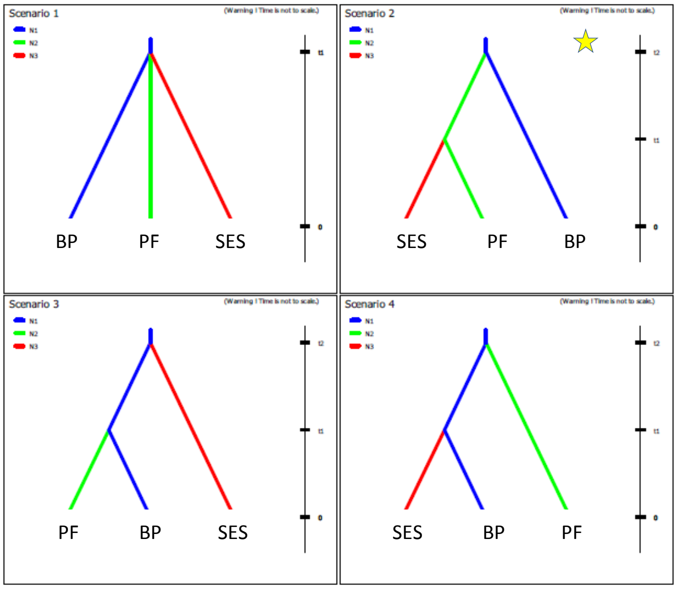

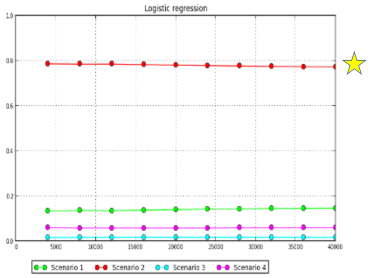

confidence in scenario choice

error		
	type I	0.296
	type II	0.232

B

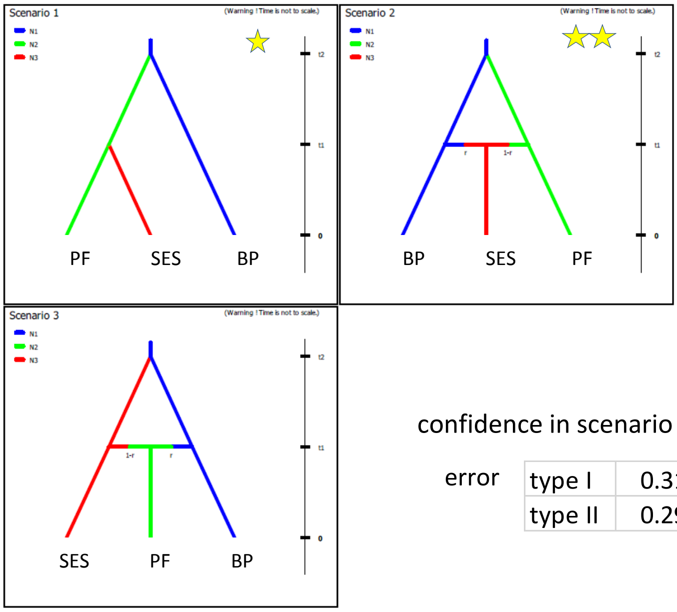

admixture		
BP:SES	r	0.189
PF:SES	1-r	0.811

confidence in scenario choice

error		
	type I	0.318
	type II	0.294

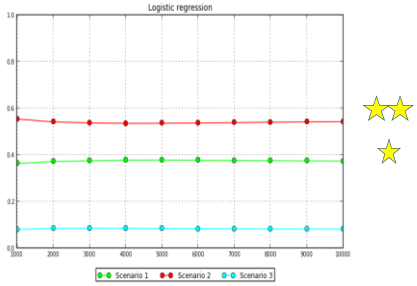

C

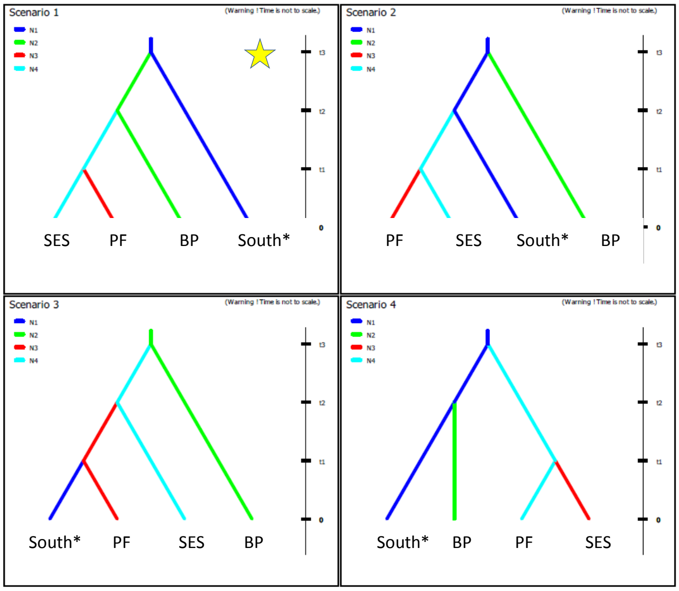

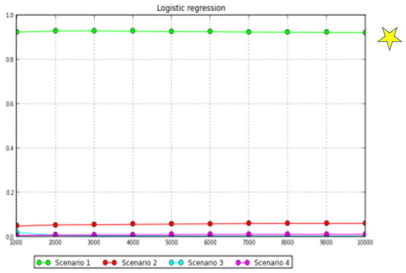

confidence in scenario choice

error		
	type I	0.388
	type II	0.16

Figure S4 cont.

D

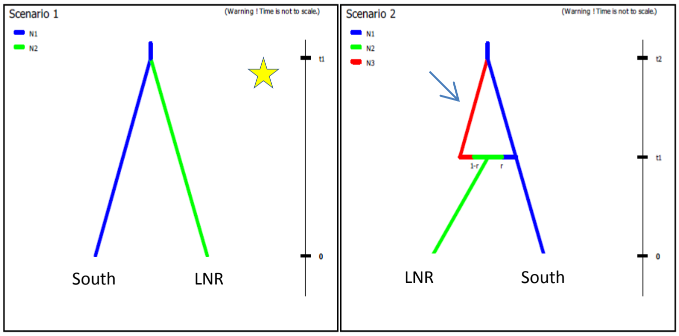

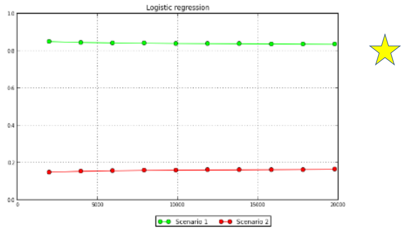

confidence in scenario choice

error		
	type I	0.176
	type II	0.331

E

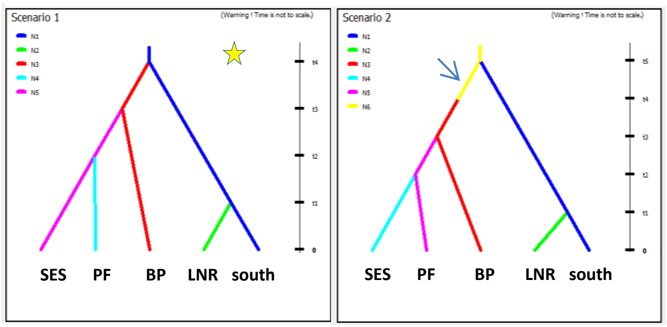

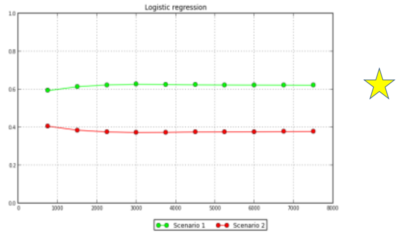

confidence in scenario choice

error		
	type I	0.412
	type II	0.359
