# Supplementary material for: Islands within an island: Population genetic structure of the endemic Sardinian newt, Euproctus platycephalus
Source: Ecol Evol. 2017 Jan 25;7(4):1190–211. doi: 10.1002/ece3.2665 (PMC5306002; doi:10.1002/ece3.2665)
Supplement: Supplementary file 5 [file ECE3-7-1190-s005.pdf]

Figure S5

A

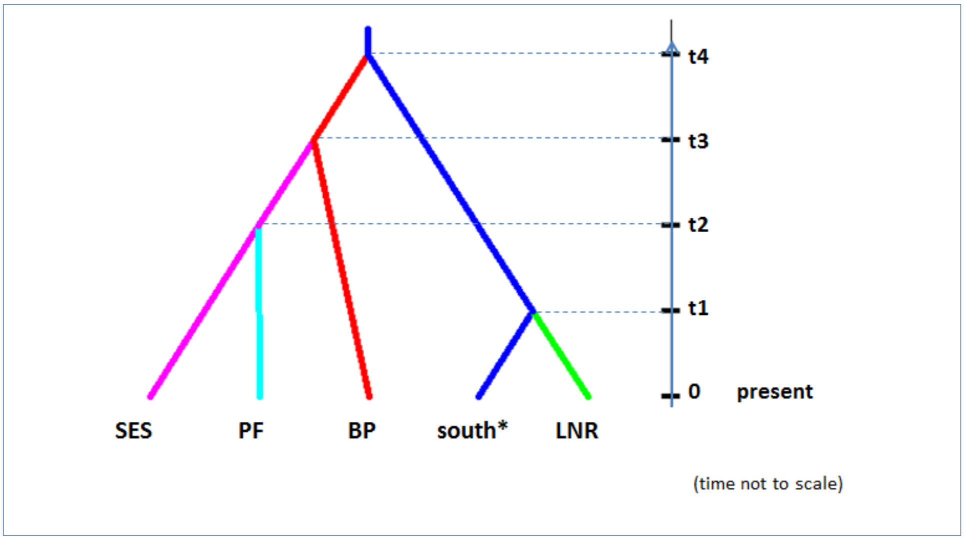

B

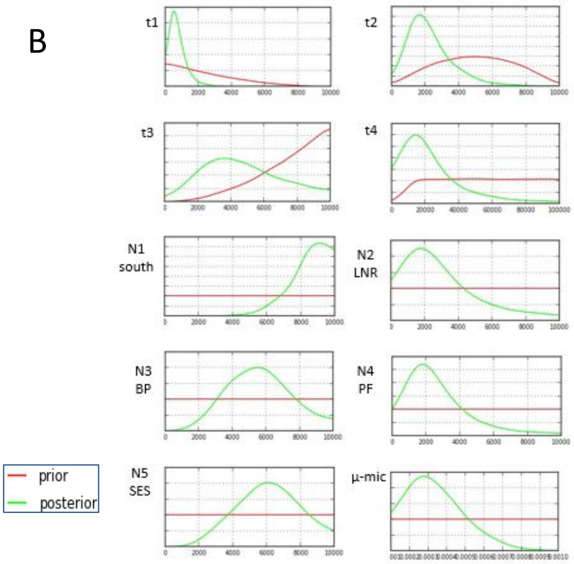

C

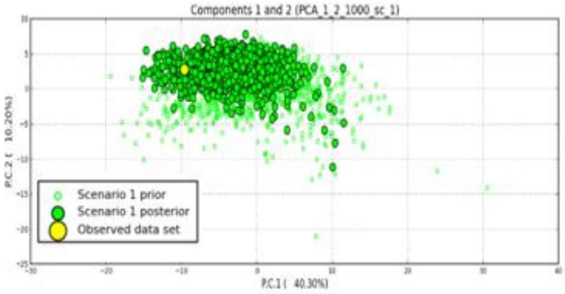

Outliers	total summary stats = 100				
< 0.05	*	15	> 0.95	*	5
	**	0		**	0
	***	0		***	0

D

parameter	type	Prior	Posterior				Measure of performance					
			median	mode	q050	q950	true value	Median	mode	RRMIS E	RMedA d	Factor 2
N1 (south)	N	U [10,10^4]	8.41 e+3	8.86 e+3	5.74 e+3	9.82 e+3	8.161 e+3	7.901 e+3	8.214 e+3	0.287	0.151	0.994
N2 (LNR)	N	U [10,10^4]	2.34 e+3	1.52 e+3	7.66 e+2	7.12 e+3	3.012 e+3	4.068 e+3	3.782 e+3	1.475	0.475	0.78
N3 (BP)	N	U [10,10^4]	4.79 e+3	4.33 e+3	2.19 e+3	8.51 e+3	5.074 e+3	5.185 e+3	5.065 e+3	0.529	0.281	0.972
N4 (PF)	N	U [10,10^4]	2.33 e+3	1.54 e+3	7.37 e+2	7.10 e+3	2.994 e+3	3.290 e+3	2.915 e+3	1.093	0.409	0.892
N5 (SES)	N	U [10,10^4]	7.06 e+3	7.58 e+3	3.94 e+3	9.43 e+3	6.943 e+3	6.948 e+3	7.093 e+3	0.393	0.216	0.988
t1	T	U [10,10^4]	8.47 e+2	4.99 e+2	2.77 e+2	3.55 e+3	1.196 e+3	1.806 e+3	1.254 e+3	3.241	0.6	0.664
t2	T	U [10,10^4]	1.57 e+3	1.45 e+3	5.12 e+2	4.08 e+3	1.801 e+3	2.046 e+3	1.543 e+3	1.471	0.489	0.766
t3	T	U [10,10^4]	4.38 e+3	2.95 e+3	1.61 e+3	8.64 e+3	4.677 e+3	5.364 e+3	4.958 e+3	0.902	0.432	0.9
t4	T	U [10,10^5]	1.38 e+4	9.19 e+3	4.26 e+3	6.20 e+4	2.081 e+4	2.723 e+4	2.114 e+4	2.011	0.565	0.728
Aμmic	M	standard	3.23 e-4	2.57 e-4	1.49 e-4	6.17 e-4	3.542 e-4	4.321 e-4	3.954 e-4	0.808	0.354	0.892
pmic	M	standard	2.39 e-1	3.00 e-1	1.34-1	3.00 e-1	2.285 e-1	1.900 e-1	1.795 e-1	0.352	0.245	0.994
snimic	M	standard	2.67 e-7	1.28 e-8	1.50 e-8	3.47 e-6	8.003 e-7	4.094 e-7	9.313 e-8	31.889	0.896	0.34
