# Supplementary material for: Islands within an island: Population genetic structure of the endemic Sardinian newt, Euproctus platycephalus
Source: Ecol Evol. 2017 Jan 25;7(4):1190–211. doi: 10.1002/ece3.2665 (PMC5306002; doi:10.1002/ece3.2665)
Supplement: Supplementary file 6 [file ECE3-7-1190-s006.pdf]

# Figure S6

A

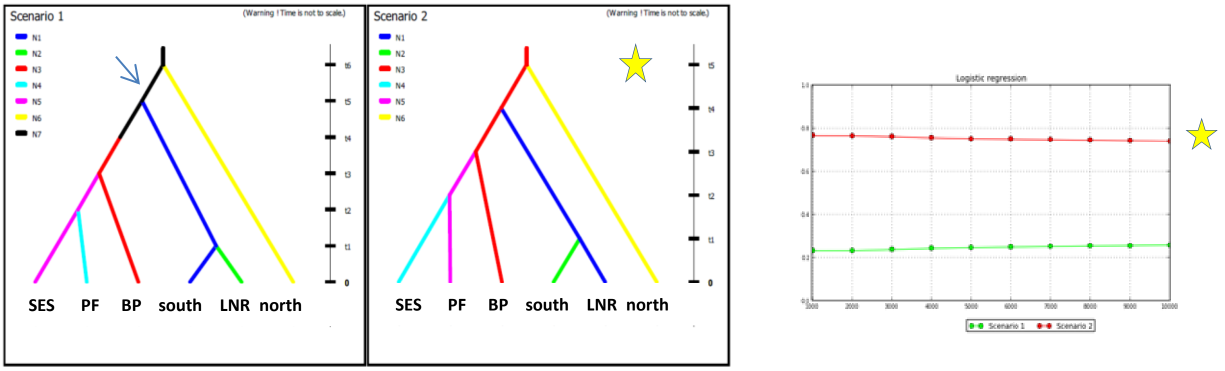


B

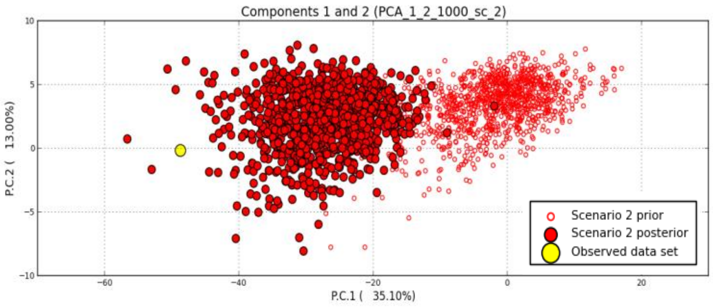


| outliers | | total summary stats = 144 | | | |
|---|---|---|---|---|---|
| < *0.05* | * | 26 | > 0.95 | * | 15 |
| | ** | 17 | | ** | 13 |
| | *** | 3 | | *** | 0 |

C

| time | event | supported scenario posterior | south/central scenarios mean (95% CI) | **n** |
|---|---|---|---|---|
| t1 | split LNR from south | 3.96 $x10^3$ | 0.93 $x10^3$ (0.41 - 1.44 $x10^3$) | 5 |
| t2 | split PF from SES | 3.44 $x10^3$ | 1.48$x10^3$ (1.16 - 1.79 $x10^3$) | 8 |
| t3 | split SES/PF from BP | 6.09 $x10^3$ | 3.91 $x10^3$ (2.77 - 5.06 $x10^3$) | 8 |
| t4 | split central from south | 5.82 $x10^4$ | 1.70 $x10^4$ (1.43 - 1.96 $x10^3$) | 4 |
| t5 | split north from central/south | 1.29 $x10^4$ | NA | NA |
| μ-mic | | 1.85 $x10^{-4}$ | 3.16 $x10^{-4}$ (3.02 - 3.31 $x10^{-4}$) | 16 |

D

| μ-mic $x10^{-4}$ | n | median | mean | range | sd |
|---|---|---|---|---|---|
| south/central | 16 | 3.22 | 3.16 | 2.29 - 3.49 | 0.268 |
| north alone | 5 | 3.52 | 3.78 | 2.99 - 3.79 | 0.392 |
| north/south/central | 7 | 1.32 | 1.36 | 1.13 - 1.85 | 0.189 |

| comparison of mean μ-mic | W | p-value |
|---|---|---|
| south/central vs north alone | 29.5 | 0.409 |
| south/central vs north/south/central | 112 | 0.00021 |
| north alone vs north/south/central | 35 | 0.0025 |
