# Supplementary material for: Islands within an island: Population genetic structure of the endemic Sardinian newt, Euproctus platycephalus
Source: Ecol Evol. 2017 Jan 25;7(4):1190–211. doi: 10.1002/ece3.2665 (PMC5306002; doi:10.1002/ece3.2665)
Supplement: Supplementary file 7 [file ECE3-7-1190-s007.pdf]

Figure S7

A

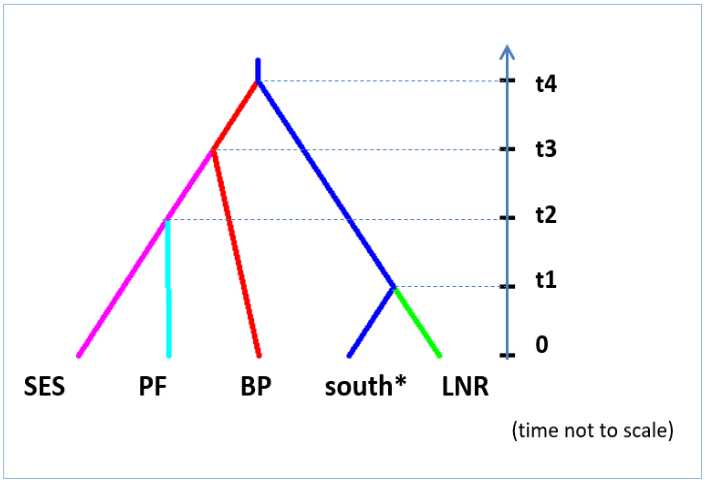

B

population	n	mean x10^3	95% CI x10^3
south	11	7.960	7.240-8.680
LNR	11	3.204	2.333-4.075
BP	14	5.068	4.512-5.624
PF	14	2.363	2.057-2.669
SES	14	5.799	5.051-6.548
north	5	5.226	3.170-7.290

C

time	event	population	bottleneck	stable
**t4**	ancestral split; south from centre	**centre**	0.6161 [0.5663, 0.6359]	0.3839 [0.3641, 0.4037]
	1.70 x 10^4 (1.43-1.96 x 10^4)	**south/LNR**	0.7240 [0.6976, 0.7503]	0.2760 [0.2497, 0.3024]
**t3**	within centre split 1	**BP**	0.2658 [0.2323, 0.2993]	0.7342 [0.7007, 0.7677]
	3.91 x10^3 (2.77-5.06 x 10^3)	**SES/PF**	0.3270 [0.2904, 0.3639]	0.6730 [0.6363, 0.7096]
**t2**	within centre split 2	**SES**	0.2678 [0.2393, 0.2963]	0.7322 [0.7037, 0.7607]
	1.47 x10^3 (1.16-1.79 x10^3)	**PF**	0.2716 [0.2571, 0.2862]	0.7285 [0.7138, 0.7429]
**t1**	within south split	**south**	0.2101 [0.1845, 0.2357]	0.7899 [0.7643, 0.8155]
	0.92 x10^3 (0.41-1.44 x10^3)	**LNR**	0.1159 [0.1079, 0.1239]	0.8841 [0.8761, 0.8921]

D

	bottleneck	stable
**north**	0.6988 [0.6641, 0.7336]	0.3012 [0.2664, 0.3359]
**centre**	0.5014 [0.4879, 0.5150]	0.4986 [0.4850, 0.5121]
**south**	0.6553 [0.6410, 0.6696]	0.3447 [0.3304, 0.3590]
	**expansion**	**stable**
**north**	0.3507 [0.2796, 0.4218]	0.6493 [0.5782, 0.7204]
**centre**	0.5087 [0.4798, 0.5375]	0.4913 [0.4625, 0.5202]
**south**	0.5435 [0.5122, 0.5741]	0.4565 [0.4253, 0.4878]

E

scenario	parameter	priors	north	central	south
**bottleneck**	**t-bn**	U [10^3,10^5]	4.31 x10^3 (1.19x10^3,4.43x10^4)	4.69 x10^4 (6.28x10^3, 9.39x10^4)	1.71 x10^4 (1.76x10^3, 7.95x10^4)
	**db**	U [10,10^3]	5.07 x10^2 (5.99x10^1,9.52x10^2)	4.99 x10^2 (3.31x10^1,9.48x10^2)	4.60 x10^2 (5.40x10^1,9.28x10^2)
	**Ne bn**	U [10,10^2]	5.22x10^1 (1.40x10^1,9.51x10^1)	5.44x10^1 (1.41x10^1,9.55x10^1)	5.73x10^1 (1.47x10^1,9.55x10^1)
**expansion**	**t-exp**	U [10^3,10^6]	4.23x10^5 (4.44x10^4,9.49x10^5)	4.81x10^5 (5.28x10^4,9.23x10^5)	5.57x10^5 (9.71x10^4,9.59x10^5)
	**de**	U [10,10^4]	5.18 x10^3 (5.99x10^2,9.46x10^3)	4.82 x10^3 (5.99x10^2,9.51x10^3)	5.64 x10^3 (4.57x10^2,9.58x10^3)
	**Ne exp**	U [10^4,10^8]	4.47x10^7 (4.60x10^6,9.33x10^7)	4.41x10^7 (3.59x10^6,9.50x10^7)	4.63x10^7 (4.72x10^6,9.49x10^7)
