# Supplementary material for: Islands within an island: Population genetic structure of the endemic Sardinian newt, Euproctus platycephalus
Source: Ecol Evol. 2017 Jan 25;7(4):1190–211. doi: 10.1002/ece3.2665 (PMC5306002; doi:10.1002/ece3.2665)
Supplement: Supplementary file 8 [file ECE3-7-1190-s008.docx]

| **mtDNA datasets** | Single sample statistics | Two sample statistics |
| --- | --- | --- |
| For simulations | 1. number of distinct haplotypes | 1. number of distinct haplotypes in pooled sample |
|  | 1. number of segregating sites | 1. number of segregating sites in pooled sample |
|  | 1. mean pairwise difference | 1. mean of within sample pairwise differences (1) |
|  | 1. variance of the number of pairwise differences |  |
|  | 1. Tajima's D statistics (2) |  |
| Model checking | 1. Number of private segregating sites | 1. mean of between sample pairwise differences (3) |
|  | 1. Mean of numbers of rarest nucleotide at segregating sites | 1. F_ST_ between two samples (4) |
|  | 1. Variance of numbers of rarest nucleotide at segregating sites |  |
|  |  |  |
| **Microsatellite datasets** | Single sample statistics | Two sample statistics |
| For simulations | 1. mean number of alleles across loci | 1. mean number of alleles across loci (two samples) |
|  | 1. mean gene diversity across loci (5) | 1. mean gene diversity across loci (two samples) |
|  | 1. mean allele size variance across loci | 1. mean allele size variance across loci (two samples) |
|  |  | 1. F_ST_ between two samples (7) |
| Model checking | 1. mean M index across loci (6) | 1. mean index of classification (two samples) (8) |
|  |  | 1. shared allele distance between two samples (9) |
|  |  | 1. (δµ)^2^ distance between two samples (10) |

**Table S1**

**Summary statistics used for mtDNA and microsatellite datasets**

1. Weir & Cockerham (1984) 2. Tajima (1989) 3. Rannala & Mountain (1997); Pascual et al (2007) 4.Hudson et al (1992)

5. Nei (1987) 6. Garza and Williamson (2001); Excoffier et al (2005) 7. Weir & Cockerham, (1984)

8. Rannala & Moutain (1997); Pascual et al (2007) 9. Chakraborty & Jin (1993) 10. Goldstein et al (1995)
